# Pan-cancer analysis of NFE2L2 mutations identifies a subset of lung cancers with distinct genomic and improved immunotherapy outcomes

**DOI:** 10.1186/s12935-023-03056-9

**Published:** 2023-10-04

**Authors:** Kewei Wang, Zixi Li, Ying Xuan, Yong Zhao, Chao Deng, Meidan Wang, Chenjun Xie, Fenglai Yuan, Qingfeng Pang, Wenjun Mao, Dongyan Cai, Zhangfeng Zhong, Jie Mei

**Affiliations:** 1 Institute of Integrated Traditional Chinese and Western Medicine, Affiliated Hospital of Jiangnan University, Wuxi, China; 2 Department of Physiopathology, Wuxi School of Medicine, Jiangnan University, Wuxi, China; 3 Department of Thoracic Surgery, Affiliated Hospital of Jiangnan University, Wuxi, China; 4 Department of Thoracic Surgery, The Affiliated Wuxi People’s Hospital of Nanjing Medical University, No. 299 Qingyang Road, Wuxi, 214023 China; 5 Department of Oncology, Affiliated Hospital of Jiangnan University, 200 Huihe Road, Wuxi, 214122 China; 6 Macao Centre for Research and Development in Chinese Medicine, Institute of Chinese Medical Sciences, University of Macau, Macao, 999078 SAR China; 7 Department of Oncology, The Affiliated Wuxi People’s Hospital of Nanjing Medical University, No. 299 Qingyang Road, Wuxi, 214023 China

**Keywords:** NFE2L2, NSCLC, Biomarker, NGS, Immunotherapy

## Abstract

**Background:**

Mutations in the KEAP1-NFE2L2 signaling pathway were linked to increased tumorigenesis and aggressiveness. Interestingly, not all hotspot mutations on NFE2L2 were damaging; some even were activating. However, there was conflicting evidence about the association between NFE2L2 mutation and Nrf2-activating mutation and responsiveness to immune checkpoint inhibitors (ICIs) in non-small cell lung cancer (NSCLC) and other multiple cancers.

**Methods:**

The study with the largest sample size (n = 49,533) explored the landscape of NFE2L2 mutations and their impact response/resistance to ICIs using public cohorts. In addition, the in-house WXPH cohort was used to validate the efficacy of immunotherapy in the NFE2L2 mutated patients with NSCLC.

**Results:**

In two pan-cancer cohorts, Nrf2-activating mutation was associated with higher TMB value compared to wild-type. We identified a significant association between Nrf2-activating mutation and shorter overall survival in pan-cancer patients and NSCLC patients but not in those undergoing ICIs treatment. Similar findings were obtained in cancer patients carrying the NFE2L2 mutation. Furthermore, in NSCLC and other cancer cohorts, patients with NFE2L2 mutation demonstrated more objective responses to ICIs than patients with wild type. Our in-house WXPH cohort further confirmed the efficacy of immunotherapy in the NFE2L2 mutated patients with NSCLC. Lastly, decreased inflammatory signaling pathways and immune-depleted immunological microenvironments were enriched in Nrf2-activating mutation patients with NSCLC.

**Conclusions:**

Our study found that patients with Nrf2-activating mutation had improved immunotherapy outcomes than patients with wild type in NSCLC and other tumor cohorts, implying that Nrf2-activating mutation defined a distinct subset of pan-cancers and might have implications as a biomarker for guiding ICI treatment, especially NSCLC.

**Supplementary Information:**

The online version contains supplementary material available at 10.1186/s12935-023-03056-9.

## Background

Currently, immune-checkpoint blocking (ICB) therapy, such as CTLA-4 or PD-1/PD-L1 inhibitors, has a therapeutic application on the majority of human tumors, and identifying individuals who are susceptible to ICB is the focus of non-surgical cancer treatment [1]. Meanwhile, some biomarkers thought to predict ICB therapeutic efficacy in various cancers include tumor mutation burden (TMB), copy number changes (CNA), microsatellite instability (MSI), immune microenvironment (TME), type and amount of T cell infiltration, and unique signaling pathways [2–6]. Furthermore, a single gene mutation is being explored as a biomarker for ICB across multiple cancer types [7–9]. Mutations in specific genes may provide the basis for therapeutic applications and immunotherapy and indicate how to proceed for their combined therapy [9, 10].

The KEAP1/NFE2L2 signaling pathway is considered one of the most crucial system components for cell defense against oxidative stress injury [11]. Once the KEAP1/NFE2L2 pathway is inappropriately active in tumor cells, it can stimulate tumor growth [12]. A previous study of 1391 non-small cell lung cancer (NSCLC) patients revealed that patients with KEAP1/NFE2L2 mutation are incredibly heterogeneous [13]. Both mutations are associated with different pathological symptoms and commonly coexist with other tumor-related mutations. In addition, NFE2L2 mutations can predict chemotherapy resistance for NSCLC [13]. Because KEAP1 is an NFE2L2 suppressor and an E3 ubiquitin ligase that tags Nrf2 for degradation, damaging mutations in both KEAP1 and NFE2L2 (Nrf2) would have opposite effects. Damaged KEAP1 leads to constitutive active Nrf2 signaling, which produces antioxidants, whereas damaging NFE2L2 mutations deactivate the Nrf2 pathway, making cells more susceptible to reactive oxygen species (ROS) [12, 14]. NFE2L2 mutation causes several types of cancer such as esophageal squamous cell carcinoma, lung squamous cell carcinoma (LUSC), head and neck cancer, prostate cancer, hepatocellular cancer, oral cancer, brain lower grade glioma, and bladder cancer [14–20].

Notedly, any two subtypes of the catenin-yaps127a-l30p/R34P mutants of NFE2L2 in hepatoblastoma of children are tumorigenic without relying on the activity of KEAP1, providing direct proof of NFE2L2 being an oncoprotein [21]. The Food and Drug Administration has granted sapanisertib (mTOR1/2 inhibitor) to NFE2L2 mutated patients with LUSC who have received platinum-based chemotherapy and ICB, regardless of KEAP1 mutations [22]. These findings suggest ICB therapy may benefit cancers with a simple NFE2L2 mutation. Meanwhile, a study shows that Chinese cancer patients, particularly NSCLC patients, with NFE2L2 mutation, can benefit more from ICIs treatment than other treatments including chemo- and radiotherapies [23]. Despite ICI treatment, a study comprising 69 samples reported that lung cancer patients with KEAP1/NFE2L2 mutation had shorter overall survival (OS) than wild-type patients [24]. The inconsistent results of two studies on the efficacy of NFE2L2 mutations on immunotherapy may be owing to limited sample sizes, insufficient tumor types and ethnic variations. In addition, several hotspot mutations including G31, E79, T80, G81, D77 and E82 on NFE2L2 are actually activating rather than damaging [25, 26]. However, the impact of these NFE2L2 activating hotspots on NSCLC immunotherapy was unclear. Moreover, after considering the fact that mechanistically KEAP1 damaging mutations and copy number loss, as well as NRF2 activating mutations and copy number gain, have identical effects on transcriptional regulation, we have consolidated these occurrences under the term Nrf2-activating mutations [27].

Herein, we sought to examine the landscape of NFE2L2 mutations and Nrf2-activating mutations, in 49,533 patients in a pan-cancer cohort (OrigiMed cohort) and the other two pan-cancer cohorts (the Memorial Sloan Kettering-Metastatic Events and Tropisms [MSK MetTropism] and the cancer genome atlas [TCGA] cohorts) and their association with clinical outcomes [28]. Moreover, we used our in-house WXPH cohort and seven publicly ICB cohorts including DFCI, MSK, MSK 1661, Pender, OAK, POPLAR and PUCH cohorts to explore the effect of NFE2L2 mutation and Nrf2-activating mutations on prognosis and response to ICIs [2, 6, 29–37]. The impact of NFE2L2 mutations and Nrf2-activating mutations on immunotherapy in NSCLC patients from the seven publicly available ICB cohorts was also evaluated. Finally, we used a dataset of 954 NSCLC patients with transcriptome data to examine the effect of NFE2L2 mutations and Nrf2-activating mutations on signaling pathway and TME alterations.

## Methods

### Study design and patients

We used ten public cohorts and one in-house cohort to explore the landscape of NFE2L2 mutations and Nrf2-activating mutations and their impact on response/resistance to ICIs in NSCLC and other solid tumors (Additional file 1: Fig. S1). NSCLC in this study covered lung adenocarcinoma (LUAD) and LUSC.

**Fig. 1:**
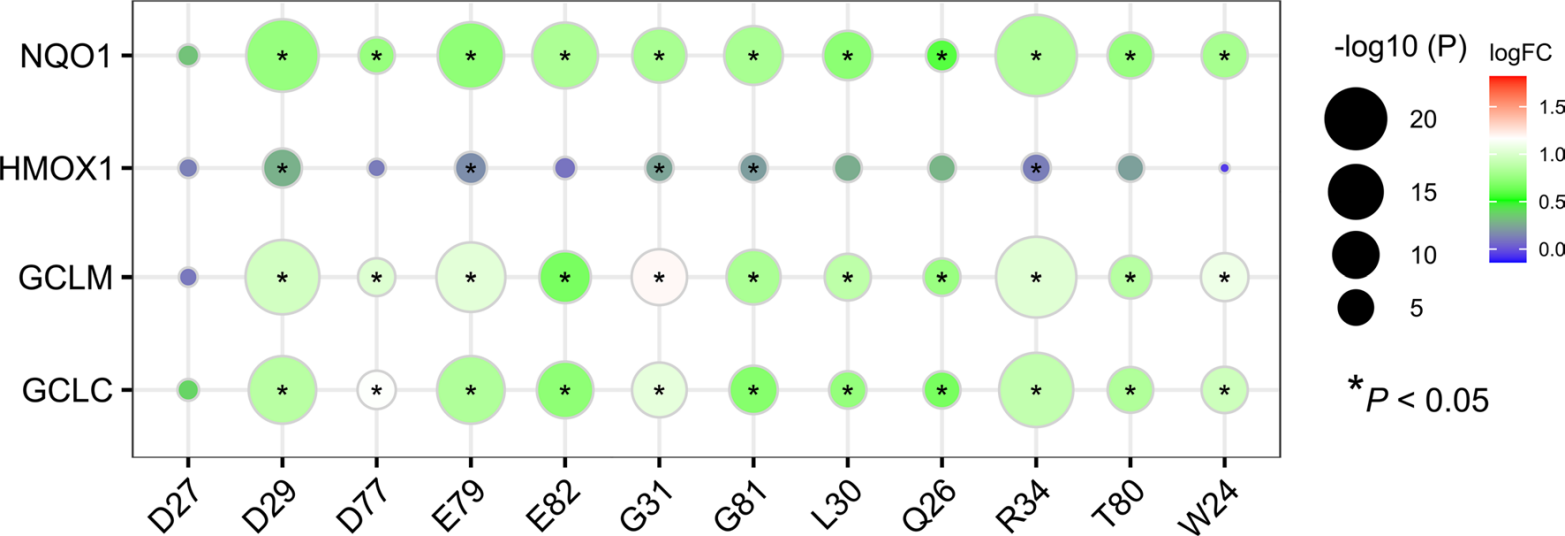
The mutation site on NFE2L2 affecting the downstream expression of key genes, including NQO1, HMOX1, GCLM, and GCLC

The damaging mutations in KEAP1 or NFE2L2 were classified based on the severity of their impact, including nonsense mutations, frame-shift indels, splice site mutations, missense mutations, and inframe indels. Additionally, we utilized pan-cancer transcriptome data from TCGA to examine the effects of several common mutation hotspots on NFE2L2, namely D27, D29, D77, E79, E82, G31, G81, L30, Q26, R34, T80, and W24, on the mRNA levels of downstream key genes NQO1, HMOX1, GCLM, and GCLC. It was observed that with the exception of D27, the remaining 11 mutation sites all resulted in increased expression levels of NQO1, HMOX1, GCLM, and GCLC (Fig. 1). Hence, these clusters of mutations were referred to as NRF2-activating mutations. Consequently, this study defined three groups: the Nrf2-activating group, which encompassed KEAP1 damaging mutations/copy number loss and NRF2-activating mutations/copy number gain; the unknown missense mutation of NFE2L2 group; and the Nrf2-inactivating group, which included damaging mutations on NFE2L2.

### TMB analysis

The calculation of TMB involved quantifying the cumulative count of nonsynonymous somatic, coding, base substitution, and indel mutations per megabase (Mb) of the genome under investigation [38]. The TMB score for each tumor sample in the OrigiMed cohort was determined by quantifying the somatic single nucleotide variations (SNVs) and insertions/deletions (InDels) per megabase (Mb) within the targeted coding area of the genome, as described in reference [39]. The count did not encompass noncoding mutations, hotspot mutations, and known germline polymorphisms that were documented in the Single Nucleotide Polymorphism Database (dbSNP) of the U.S. National Center for Biotechnology Information. To measure TMB in the TCGA cohort, genomic data is analyzed to identify mutations in the tumor samples. The total number of nonsynonymous somatic, coding, base substitution, and indel mutations is counted, and this count is divided by the size of the target region to obtain TMB per megabase (Mb) [40]. The captured region lengths for samples sequenced using the MSK-IMPACT panel were reported as 0.98, 1.06, and 1.22 megabases (Mb) for the 341, 410, and 468 gene panels, respectively [28].

### Public cohorts

The OrigiMed cohort was comprised of 10,194 patients across 25 tumor types who underwent a next-generation sequencing (NGS) assay in the Clinical Laboratory Improvement Amendments (CLIA)-certified and College of American Pathologists (CAP)-accredited laboratory, Shanghai, China. Comprehensive genomic profiling for the Chinese cohort was performed using customized panels of 450 genes [39].

Somatic mutation data of the MSK MetTropism cohort (n = 25,766 patients across pan-cancers) and the TCGA cohort (n = 10,953 patients across pan-cancers) data were downloaded from cBioPortal platform (https://www.cbioportal.org/) [41] The transcriptomic data of 954 NSCLC patients from TCGA cohort was obtained from Genomic Data Commons (GDC) Data Portal (https://portal.gdc.cancer.gov/).

The efficacy of ICIs was assessed in seven publicly ICB cohorts including DFCI, MSK, MSK 1661, Pender, OAK, POPLAR and PUCH cohorts [2, 6, 29–37]. The MSK 1661 cohort was composed of 1661 patients from various tumor types who were treated with ICIs and had clinical and somatic mutation data (https://www.cbioportal.org/). The DFCI cohort included 176 patients who were being treated with ICIs for bladder cancer, lung cancer, or melanoma. [5, 6]. The POPLAR and OAK cohorts encompassed 429 NSCLC patients with blood-based next-generation sequencing data [31]. The Pender cohort had 75 patients across nine cancer types [30]. In the PUCH cohort, 91 patients with gastrointestinal cancer were treated by ICIs [29]. The MSK cohort was a merged cohort of 97 patients with melanoma or NSCLC from cBioPortal platform [42, 43].

### In-house cohort

The WXPH cohort consisted of 65 NSCLC patients who were treated with anti-PD-1/PD-L1 agents and NGS tests and were recruited by the Affiliated Wuxi People's Hospital of Nanjing Medical University between 2018 and 2022. Burning Rock Dx performed the PD-L1 and NGS testing. The Clinical Research Ethics Committee of the Affiliated Wuxi People's Hospital of Nanjing Medical University granted ethical approval (Number: KY21126) for the collection of these participants.

### Statistical analysis

The t-test or Mann–Whitney test were used to compare differences between the two groups. To compare categorical variables, the Chi-square tests or Fish exact probabilities were utilized. Pearson or Spearman correlation analysis was used to directly measure the relationship between the two continuous variables. The legend provides the aforementioned statistical details, such as the statistical tests performed, the number of samples, dispersion, data type, and how the level of significance was calculated. The Kaplan–Meier method was implemented to make survival curves, and the Log-rank test was performed to compare differences. The Cox regression model was used to compare survival outcomes across several groups, and multiple testing correction was performed using the Bonferroni technique, with p < 0.05 considered significant. To account for confounding effects, multivariate Cox regression models from the survminer package were in use [44]. For differential gene expression (DGE) analysis, the R package DESeq2 was utilized [45]. R package Cluster-Profiler was used for gene set enrichment analysis (GSEA) in NFE2L2-WT, Nrf2-activating mutation and Nrf2-inactivating mutation patients [46]. The somatic interaction analysis was performed with the somatic interactions function of Maftools on the NFE2L2 and top 10 mutant genes [47]. Unsupervised hierarchical clustering was performed with the ComplexHeatmap R package [48]. All statistical analysis was performed with R-4.0.3. A p-value less than 0.05 is typically considered to be statistically significant.

## Results

### Nrf2-activating mutations in the OrigiMed cohort

**Fig. 2:**
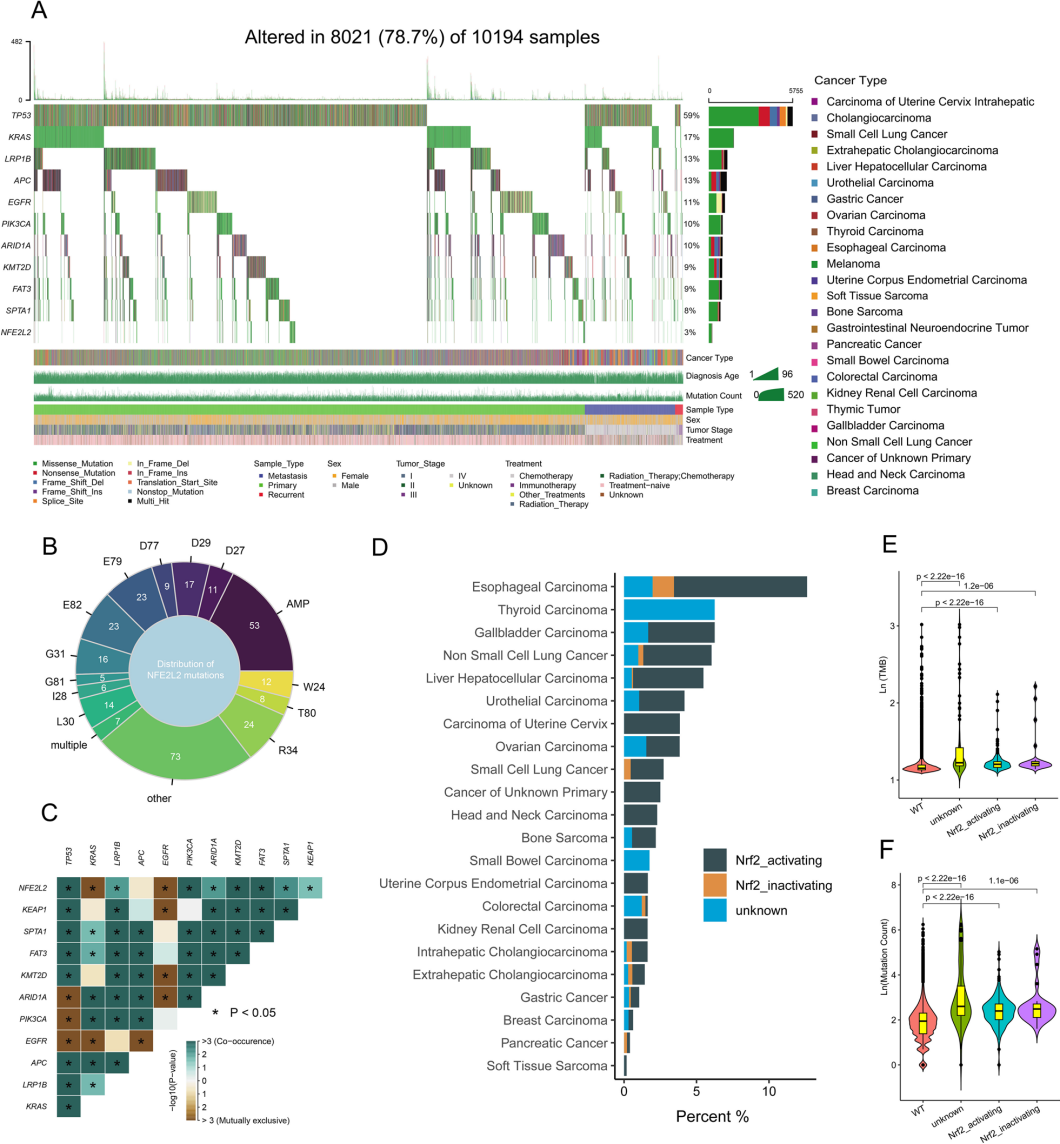
Overview of NFE2L2 mutations and Nrf2-activating mutations in the OrigiMed cohort. (**A**) The NFE2L2 mutations profiles and the clinical characters of 10,194 patients. (**B**) Distribution of NFE2L2 mutations according to locations of the mutation. (**C**) Co-occurrence/mutual exclusivity of NFE2L2 and other genes. (**D**) The prevalence of Nrf2-activating mutations, Nrf2-inactivating mutations and unknown NFE2L2 mutations in each cancer type. Violin plots of TMB (**E**) and mutation count (**F**) among four groups: Nrf2-activating mutations, Nrf2-inactivating mutations, unknown NFE2L2 mutations and WT groups. TMB: tumor mutation burden. WT: wide type

There were 301 NFE2L2 mutations (MUs, 3.0%) in the OrigiMed cohort with esophageal carcinoma (ESCA, 12.1%) ranking first followed by thyroid carcinoma (6.3%), gallbladder carcinoma (5.4%) and NSCLC (4.7%) (Fig. 2 A and B). The missense mutation, which generated an amino acid change and a protein change, was the most pathogenic/suspected pathogenic NFE2L2 MU (Fig. 2 A). Sev**er**al NFE2L2 MU-subtypes, such as R34 (n = 24), E79 (n = 23), D77 (n = 9), D29 (n = 17), G31 (n = 16), L30 (n = 14), D27 (n = 11), and E82 (n = 23) were clustered in identified hotspots (Fig. 2A and B). Furthermore, we retrieved the top ten mutated genes and KEAP1, and identified a concurrent pattern of NFE2L2 with KEAP1, TP53, LRP1B, PIK3CA, ARID1A, KMT2D, FAT3 and SPTA1 mutations, as well as a mutually exclusive pattern of NFE2L2 with KRAS and EGFR mutations (Fig. 2C).

Given the interaction of NFE2L2 with KEAP1 via the DLG and ETGE motifs [11], it was found that the prevalence of Nrf2-activating mutation (MU) was higher than that of Nrf2-inactivating mutation (MU) in the majority of cancer types (Fig. 2D and Additional file 1: Fig. S2A). Furthermore, compared to NFE2L2 WT patients, those with Nrf2-activating MU had a higher TMB and mutation count (Fig. 2E and F). Nrf2-activating MUs were more commonly observed in tumors obtained from male patients and those with stage II-IV while similar results were not observed for those with metastasis and different therapy methods (Additional file 1: Fig. S2B–E). Similarly, there was no significant correlation between the frequency of NFE2L2 MU and objective response rates to ICIs across various tumor types (r = − 0.067; p = 0.78; Additional file 1: Fig. S2F).

### Characteristics for Nrf2-activating mutations in the MSK MetTropism cohort

**Fig. 3:**
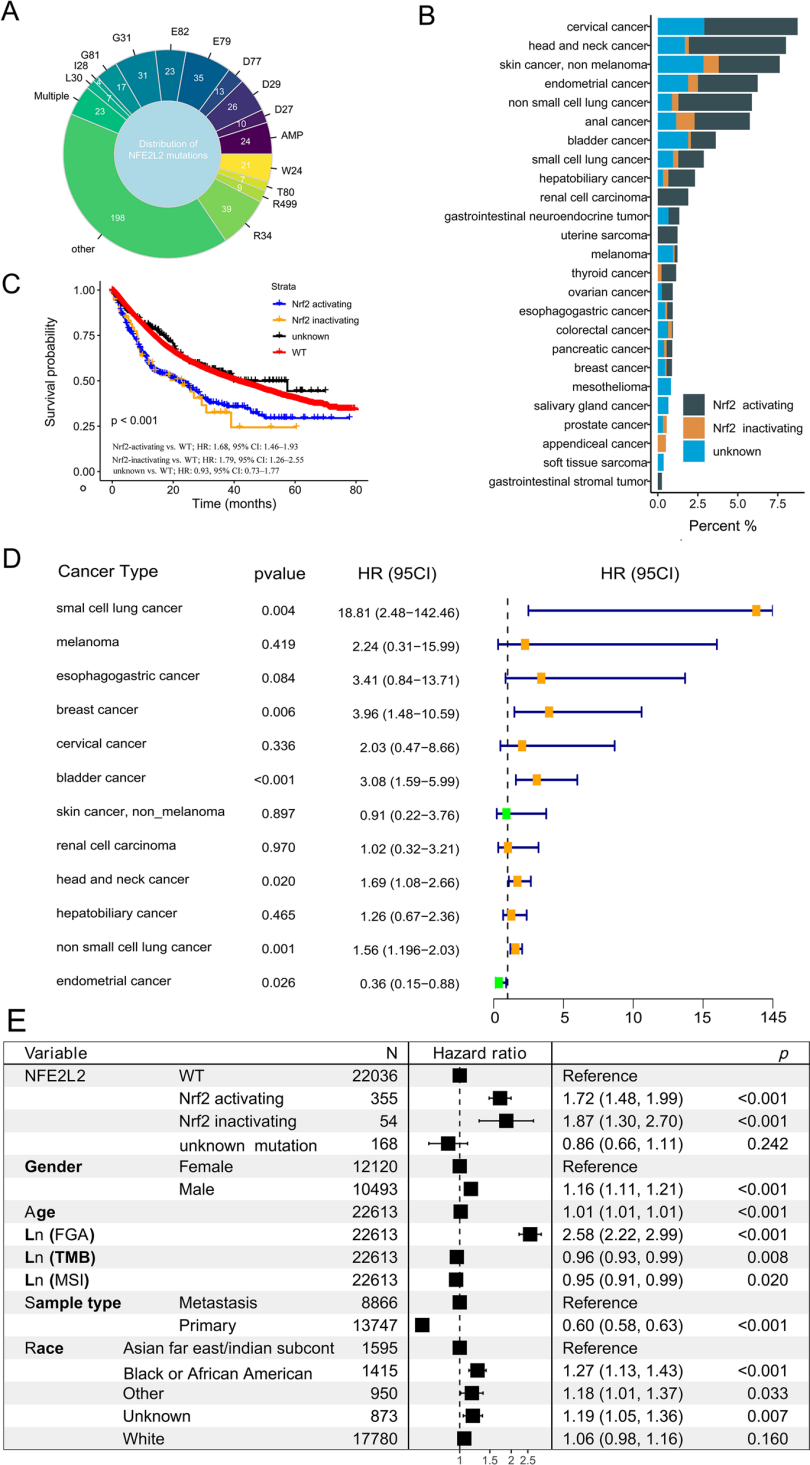
Summary of NFE2L2 Nrf2-activating mutations in the MSK MetTropism cohort. (**A**) Distribution of NFE2L2 mutations according to locations of the mutation. (**B**) The prevalence of Nrf2-activating mutations and Nrf2-inactivating mutations in each cancer type. (**C**) Kaplan–Meier survival curves of OS among Nrf2-activating mutations, Nrf2-inactivating mutations, unknown NFE2L2 mutations and WT groups. (**D**) Association of Nrf2-inactivating mutations with OS stratified by cancer type. (**E**) The multivariate Cox regression analysis for OS in patients. MSI: microsatellite instability; FGA: fraction genome altered; OS: overall survival; MU: mutation; WT: wild type

We used the MSK MetTropism cohort to evaluate the molecular characteristics of NFE2L2 MU across pan-cancers, and found that 1.9% of patients (n = 487) had at least one NFE2L2 MU (Fig. 3A and Additional file 1: Fig. S3A). There were 388 Nrf2-activating MUs and 58 Nrf2-inactivating MUs among three different Nrf2 and KEAP1 MU groups (D77 [n = 13], E79 [n = 35], E82 [n = 23], G31 [n = 31], G81 [n = 17], and T80 [n = 7]; Fig. 3A and Additional file 1: Fig. S3B). In the MSK MetTropism cohort, head and neck cancer and cervical cancer had the highest Nrf2-activating MUs frequency (6.07%), followed by cervical cancer (5.83%) and NSCLC (4.59%, Fig. 3B).

Interestingly, there was a significant association between the frequency of NFE2L2 MU and median TMB across multiple tumor types (r = 0.63, *P* < 0.001; Additional file 1: Fig. S3C). TMB has been shown to be a predictive biomarker for ICIs in various cancers, implying that cancer patients with NFE2L2 MUs may benefit from ICIs. This hypothesis further was validated by more TMB in Nrf2-activating MU group than in NFE2L2 WT group (p < 0.001, Additional file 1: Fig. S3D). Similarly, the Nrf2-activating MU group had higher mutation count, MSI score and fraction genome altered (FGA) than NFE2L2 WT group (all p < 0.001, Additional file 1: Fig. S3E–G), whereas metastatic status did not differ significantly (p = 0.61, Additional file 1: Fig. S3H). Moreover, there was a significant association between the frequency of NFE2L2 MU and objective response rates to ICIs across multiple cancer types (r = 0.46; p = 0.015; Additional file 1: Fig. S3I).

Univariate analysis revealed an association between NFE2L2 MU and shorter OS in the MSK MetTropism cohort (HR: 1.28, 95% CI 1.12–1.47; Additional file 1: Fig. S3J). In addition, survival analysis stratified by cancer type revealed that NFE2L2 MU was associated with shorter OS in prostate cancer (HR: 3.02, 95% CI 1.25–7.30; Additional file 1: Fig. S3L) and NSCLC (HR: 1.57, 95% CI 1.28–1.94), while contrary association in endometrial cancer (HR: 0.27, 95% CI 0.13–0.58). Similarly, Univariate survival analysis revealed that Nrf2-activating MU (HR: 1.68, 95% CI 1.46–1.93) and Nrf2-inactivating MU (HR: 1.79, 95% CI 1.26–2.55) had a significant association with shorter OS for pan-cancer types (Fig. 3C).

Furthermore, survival analysis stratified by cancer type revealed that there was a significant association between the frequency of Nrf2-activating MU and shorter OS only in small cell lung cancer (HR: 18.81, 95% CI 2.48–142.46; Fig. 3D) and NSCLC (HR: 1.56, 95% CI 1.19–2.03). Similar results were found in the NSCLC patients with Nrf2-inactivating MU (HR: 1.91, 95% CI 1.11–3.30; Additional file 1: Fig. S3M). So, after controlling for other confounding factors such as sex, age, TMB, FGA, MSI, metastatic status, and race, we discovered significant association of Nrf2-activating MU and Nrf2-inactivating MU with shorter OS (HR: 1.72, 95% CI 1.48–1.99; HR: 1.87, 95% CI 1.30–2.70; Fig. 3E).

### Clinical outcomes for Nrf2-activating mutations across pan-cancer and NSCLC in TCGA cohort

**Fig. 4:**
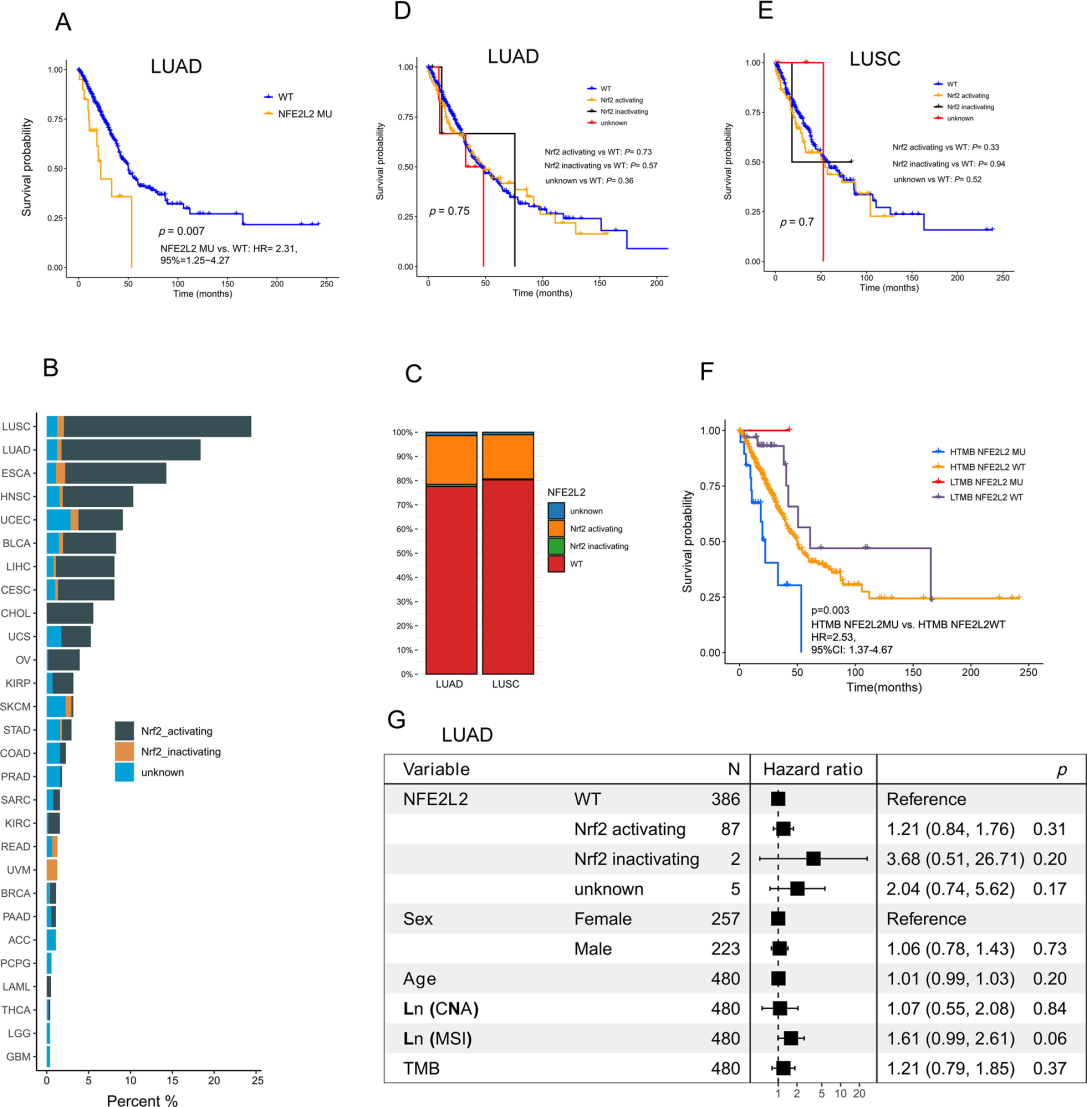
Clinical outcomes for Nrf2-activating mutations in NSCLC patients from TCGA cohort. (**A**) Kaplan–Meier survival curves of OS between NFE2L2 MU and WT groups from LUAD. datasets. (**B**) The prevalence of Nrf2-activating mutations and Nrf2-inactivating mutations in each cancer type. (**C**) The proportion of Nrf2-activating mutations and Nrf2-inactivating mutations in LUAD and LUSC. Kaplan–Meier survival curves of OS among Nrf2-activating MU, Nrf2-inactivating MU, unknown NFE2L2 MU and WT groups from LUAD (**D**) and LUSC (**E**). (**F**) Kaplan–Meier survival curves of OS among HTMB NFE2L2 MU, HTMB NFE2L2 WT, LTMB NFE2L2 MU and LTMB NFE2L2 WT patients with NSCLC from TCGA cohort. HTMB: high tumor mutation burden, L TMB: Low tumor mutation burden. (**G**) The multivariate Cox regression analysis for OS in patients with LUAD. CNA: copy number alteration; MSI: microsatellite instability; OS: overall survival

Survival analysis stratified by cancer type in TCGA dataset revealed that there was a significant association between the frequency of NFE2L2 MU and shorter OS in LGG (HR: 7.43, 95% CI 1.81–30.38) and LUAD (HR: 2.31, 95% CI 1.25–4.27; Additional file 1: Fig. S4A, Fig. 4A). Of patients harboring NFE2L2/KEAP1 mutations in TCGA cohort, LUSC (22.3%) had the highest frequency of Nrf2-activating MU, followed by LUAD (16.6%), ESCA (12.1%), HNSC (8.4%) and UCEC (6.7%), respectively (Fig. 4B).

From a total of 1053 patients diagnosed with NSCLC, 113 (20.0%) had Nrf2-activating MU in the LUAD subtype and 88 (18.0%) did so in the LUSC subtype (Fig. 4C). NSCLC patients with NFE2L2 MU did not have significantly shorter OS compared to those with WT (p = 0.97, Additional file 1: Fig. S4B). In addition, Univariate analysis showed that patients with Nrf2-activating MU did not have significantly shorter OS compared to those with WT in the LUAD (p = 0.75, Fig. 4D) and LUSC (p = 0.70, Fig. 4E) datasets from TCGA cohort. Even after controlling for copy number variation (CNA), MSI, TMB, age and gender, LUAD and LUSC patients with Nrf2-activating MU did not have significantly shorter OS compared to those with WT (*p* = 0.89, *p* = 0.31; Fig. 4G, Additional file 1: Fig. S4C). We also classified NSCLC patients into four groups based on NFE2L2 status and TMB level: high-TMB (HTMB) NFE2L2 MU, HTMB NFE2L2 WT, low-TMB (LTMB) NFE2L2 MU, and LTMB NFE2L2 WT. In HTMB group, patients with NFE2L2 MU achieved shorter OS than those with NFE2L2 WT (HR: 2.53, 95% CI 1.37 − 4.67, Fig. 4F).

### NFE2L2 MU and ICI efficacy across pan-cancers and NSCLC

**Fig. 5:**
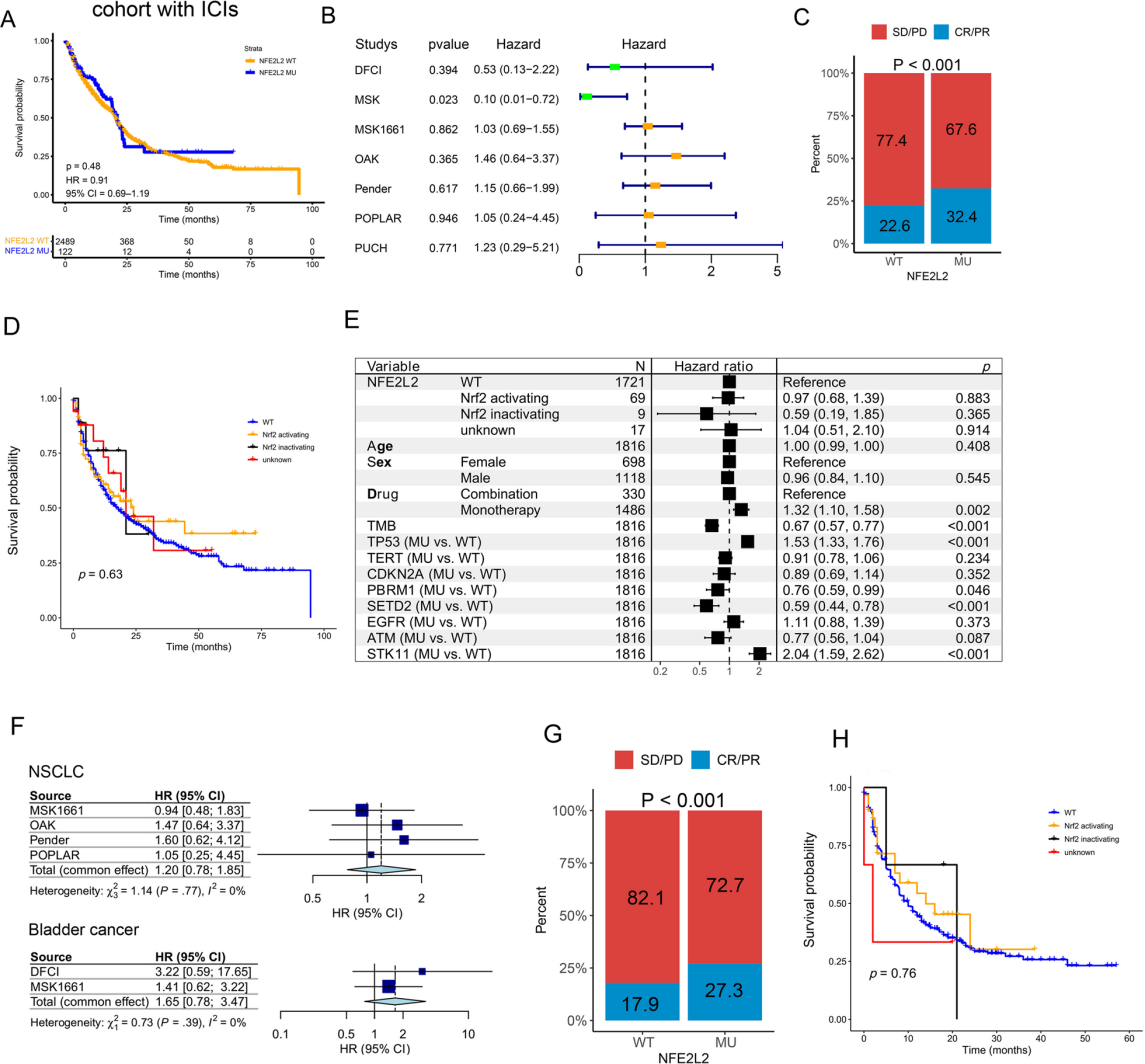
Summary of NFE2L2 mutations and survival outcomes in patients receiving ICIs. (**A**) Kaplan–Meier survival curves of OS between NFE2L2 MU and NFE2L2 WT groups in the pooled cohort composed of DFCI, MSK, MSK1661, OAK, Pender, POPLAR and PUCH cohorts. (**B**) Association of NFE2L2 mutation with OS stratified by study with ICIs treatment. The proportion of CR/PR to ICIs in NFE2L2 mutated patients from the pooled (**C**, n = 908) and NSCLC (**G**, n = 491) cohorts. Kaplan–Meier survival curves of OS among Nrf2-activating MU, Nrf2-inactivating MU, unknown NFE2L2 MU and WT groups from pan cancer (**D**) and NSCLC (**H**) with ICIs treatment. (**E)** The multivariate Cox regression analysis for OS in patients with ICIs treatment. (**F**) Meta-analysis for NSCLC and bladder cancer to summarize association of NFE2L2 mutation with OS after ICIs treatment

To investigate the predictive efficacy of NFE2L2 MU for ICI treatment in pan-cancer, we evaluated the association between NFE2L2 MU and clinical outcomes in multiple types tumors of a pooled cohort (n = 2611) composed of DFCI, MSK, MSK1661, OAK, Pender, POPLAR and PUCH cohorts. Clinical characteristics of the pooled cohort, including cancer types, NFE2L2 MU, response to ICI, gender, ICIs treatment, age and TMB were showed in Table 1. The NFE2L2 MU was not associated with shorter OS in the pooled cohort with ICI treatment (HR: 0.91, 95% CI 0.69 − 1.19; Fig. 5A). In addition, survival analysis stratified by study type showed that patients from the DFCI, MSK1661, OAK, Pender, POPLAR, and PUCH cohorts did not show any association between NFE2L2 MU and OS, however NFE2L2 MU was positively related with better OS in the MSK cohort (Fig. 5B). What piqued our curiosity was the fact that patients with NFE2L2 MU had a higher proportion of complete response (CR)/partial response (PR) to ICIs than those with WT (p < 0.001, Fig. 5C).

**Table 1:** Patient characteristics in seven cohorts with ICIs treatment

Study	n	Cancer types	NFE2L2 MU	Response to ICI (n, ORR)	Female	Male	ICIs treatment	Age^a^	TMB^b^	Mutation Count^b^
DFCI	176	Bladder cancer, HNSCC, Kidney cancer, Lung cancer	8	129(43)	62	114	CTLA-4/PD-1/PD-L1	59.7 (15.6)	NA	NA
MSK	155	Melanoma, NSCLC	9	99(56)	71	84	CTLA-4/PD-1/PD-L1 and /Chemotheragy	62.2 (11.6)	7.6 (3.5, 17.7)	105 (226, 508)
MSK1661	1661	11 cancer types	49	0(0)	627	1034	CTLA-4/PD-1/PD-L1 and /Chemotheragy	61.4 (13.7)	5.9 (2.9, 11.2)	4(6, 12)
OAK	324	NSCLC	20	257(46)	121	203	PD-1/PD-L1	63.6 (8.9)	8 (4, 15)	NA
Pender	98	18 cancer types	20	73(16)	54	44	CTLA-4/PD-1/PD-L1 and /Chemotheragy	54.4 (13.1)	7 (3, 11.3)	NA
POPLAR	105	NSCLC	12	81(16)	33	72	PD-1/PD-L1	61.3 (9.4)	7 (3.8, 17)	NA
PUCH	92	gastrointestinal cancer	4	57(35)	25	67	CTLA-4/PD-1/PD-L1	56 (12.9)	4.6 (2.5, 9.1)	NA

^a^Mean (sd, standard deviation)

^b^Median (IQR, inter quartile range)

Furthermore, there was not significant difference of over survival among four groups including Nrf2-activating MU, Nrf2-inactivating MU, unknown NFE2L2 MU and WT groups (log rank test, *P* = 0.63, Fig. 5D). Because several genes mutations such as TP53, TERT, CDKN2A, PBRM1, SETD2, EGFR, ATM, KEAP1 and STK11 were found to affect the prognosis of cancer patients[28, 49–51], we selected 1816 patients from two immunotherapy cancer cohorts including MSK cohort and MSK1661 cohort, sufficient genes mutation information and clinical characteristics to conduct multivariate Cox regression model with confounding factors adjusted, revealing no significant association of Nrf2-activating MU with shorter OS (HR: 0.97, 95% CI 0.68 − 1.39; Fig. 5E).

Following that, there was no significant association of NFE2L2 MU with OS in NSCLC (HR: 0.77, 95% CI 0.47 − 1.24; Additional file 1: Fig. S5A) or other cancer types. We also performed meta-analysis for each cancer type to summarize results from different cohorts, and found that NFE2L2 MU did not have a significant association with OS (HR: 0.94, 95% CI 0.70 − 1.26; Additional file 1: Fig. S5A). Similarly, meta-analysis for NSCLC, Bladder cancer and Melanoma revealed no significant association of NFE2L2 MU with OS (HR: 1.20, 95% CI 0.78 − 1.85; 1.65, 95% CI 0.78 − 3.47; 0.68, 95% CI 0.16 − 2.83; Fig. 5F and Additional file 1: Fig. S5A). But NSCLC patients with NFE2L2 MU had a higher proportion of CR/PR to ICIs than those with WT (Fig. 5G, p < 0.001). Therefore, we further implement survival analysis in NSCLC patients from MSK1661, OAK and POPLAR cohorts, and found that NFE2L2 MU did not have a significant association with OS (MSK1661, HR: 0.93, 95% CI 0.48 − 1.82; OAK, HR: 1.46, 95% CI 0.64–3.37; POPLAR, HR: 1.05, 95% CI 0.24 − 4.45; Additional file 1: Fig. S5B-D). Furthermore, there was not significant difference of over survival among four groups including Nrf2-activating MU, Nrf2-inactivating MU, unknown NFE2L2 MU and WT groups (log rank test, *P* = 0.76, Fig. 5H).

Given the importance of TMB in immunotherapy efficacy, we investigated the interaction of NFE2L2 MU and TMB on OS of patients receiving immunotherapy. Although patients with NFE2L2 MU had more TMB than those with NFE2L2 WT (p < 0.001, Additional file 1: Fig. S5E), and patients with HTMB had higher OS than those with LTMB (HR: 0.59, 95% CI 0.50 − 0.68; Additional file 1: Fig. S5F), NFE2L2 MU did not have different OS between LTMB (HR: 0.89, 95% CI 0.53–1.49; Additional file 1: Fig. S5G) or HTMB groups (HR: 1.39, 95% CI 0.85–2.30; Additional file 1: Fig. S5G). The findings suggested that individuals with NSCLC who have NFE2L2 MU and Nrf2-activating MU might benefit from ICIs regardless of TMB.

### NFE2L2 MU and ICI efficacy in the WXPH cohort

**Fig. 6:**
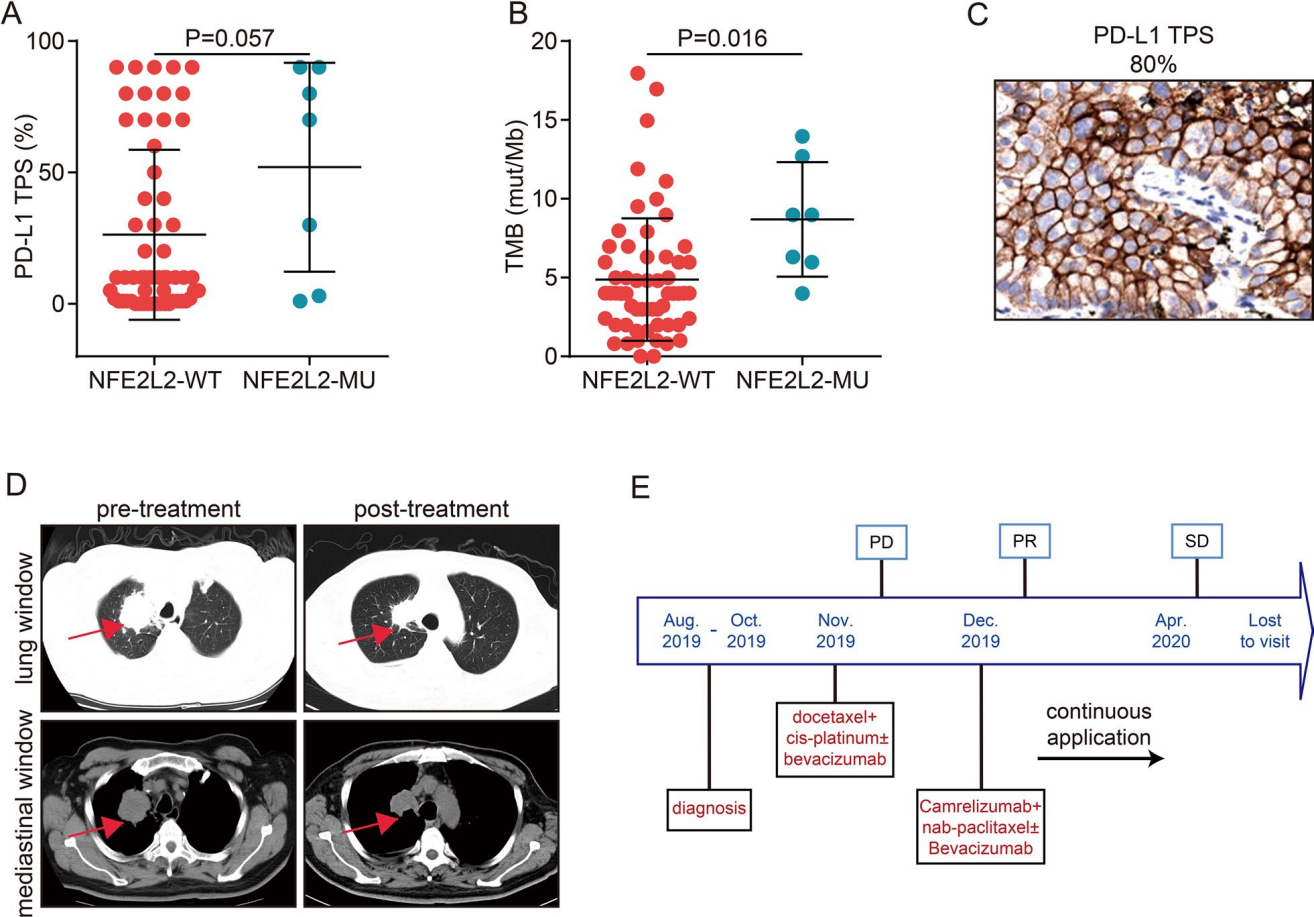
The effect of immunotherapy on NFE2L2 patients with NSCLC in the WXPH cohort. Comparison of PD-L1 TPS (**A**) and TMB (**B**) between NFE2L2 MU and NFE2L2 WT NSCLC groups in the WXPH cohort, TPS: tumor proportion score. (**C**) Immunohistochemistry showing PD-L1 expression. (**D**) Mediastinal and lung computed CT imaging of patients before and after immunotherapy. The region of the lesion was depicted by the red arrow. (**E**) Fig. 6E illustrates the treatments procedure.

Furthermore, we verified the association between NFE2L2 MU and PD-L1 expression and TMB levels in the WXPH cohort. In our study, 65 individuals were enrolled, with seven patients (10.8%) having NFE2L2 MU. Notably, NSCLC patients with NFE2L2 MU had greater PD-L1 and TMB levels as compared to patients without NFE2L2 MU (Fig. 6A and B). In addition, one of the seven patients with NFE2L2 MU underwent combined immunotherapy (camrelizumab + nab-paclitaxel + bevacizumab). This patient had elevated PD-L1 expression (TPS: 80%, Fig. 6C) and several mutations, including STK11 mutation, KEAP1 mutation, and PD-L1 amplification (Additional file 1: Table S1). After two cycles of combination immunotherapy, the therapeutic response achieved PR (Fig. 6D). Figure 6E depicts the therapy procedure.

### Signal differences between Nrf2-activating mutation and NFE2L2 WT groups in the NSCLC cohort

**Fig. 7:**
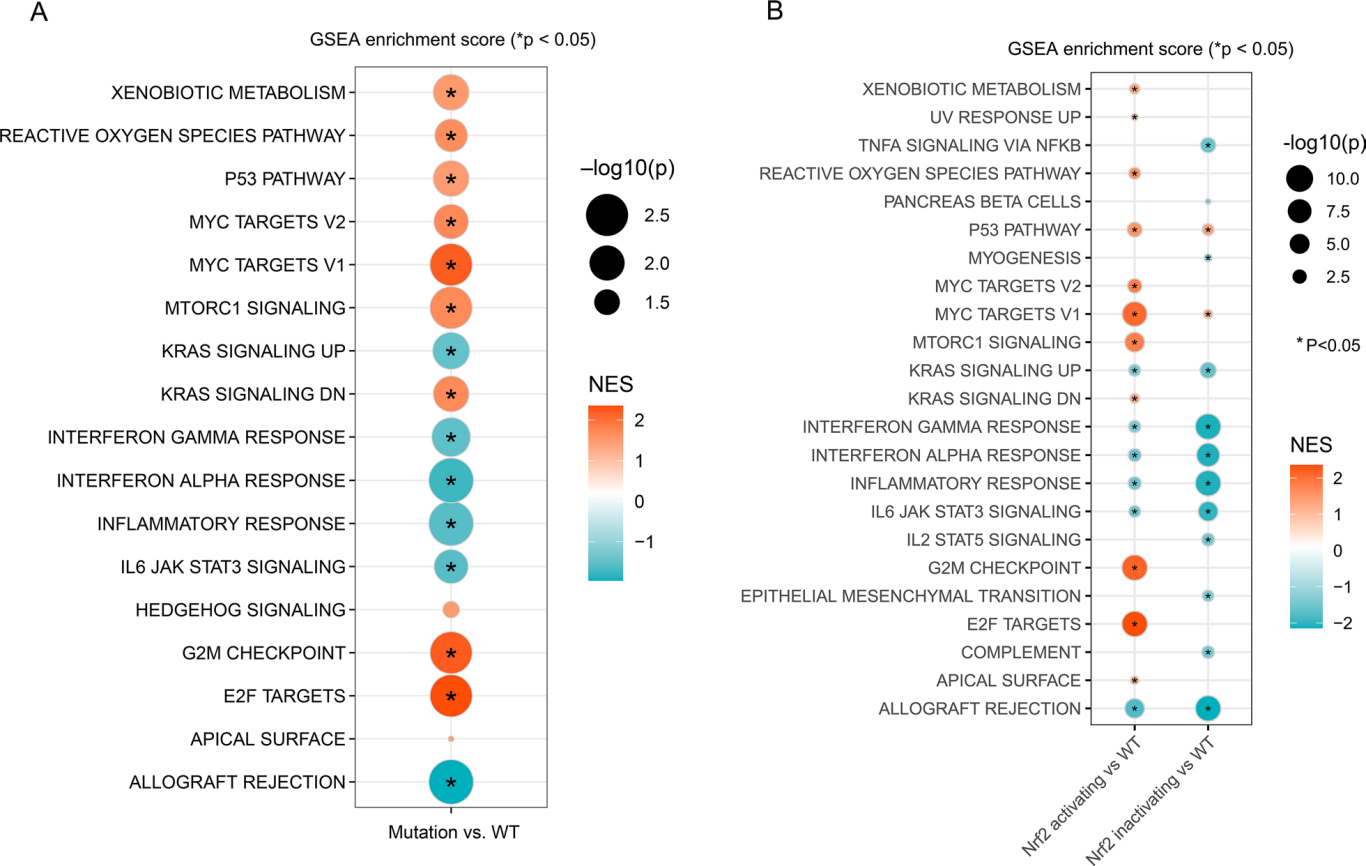
Significantly enriched signaling pathways in NFE2L2-MU (**A**), Nrf2-activating MU (**B**) and Nrf2-inactivating MU patients with NSCLC in TCGA cohort. GSEA: gene set enrichment analysis; NES: normalized enrichment scores

Since survival analysis by cancer type revealed that NSCLC patients with NFE2L2 MU had a poorer OS (Additional file 1: Fig. S3L), but not NFE2L2 mutated NSCLC patients treated with ICIs (Fig. 5F), we used TCGA NSCLC transcriptomic data to assess signal pathway alterations between NFE2L2 MU and NFE2L2 WT groups. We determined that the p53, mTORC1, and reactive oxygen species (ROS) signaling pathways were significantly activated in NFE2L2 MU group using GSEA analysis (Fig. 7A). ROS activation can trigger several downstream signal pathways, including the RAS-RAF-MEK-ERK (MAPK), PI3K-AKT-mTOR, JAK-STAT3, and VAV3-RHO pathways [52]. More than, cell cycle related pathways such as MYC targets v2, MYC targets v1 and G2M checkpoint were prevalent in NFE2L2 MU group (all P < 0.05, Fig. 7A). Similar pathways were found in the Nrf2-activating MU group (all P < 0.05, Fig. 7B). Notedly, inflammatory signaling pathways such as interferon gamma response, interferon alpha response, inflammatory response, IL6 JAK STAT3 signaling were significantly decreased in Nrf2-activating MU and Nrf2-inactivating MU groups (Fig. 7B), suggesting that Nrf2-activating and Nrf2-inactivating Mus might induce immunosuppression. Moreover, the decreased expression of BTLA, CTLA4, HAVCR2, PDCD1, TIGIT and VSIR in Nrf2-activating MU group compared to NFE2L2 WT group confirmed the immunosuppression of Nrf2-activating MU NSCLC (Additional file 1: Fig. S6). Notedly, the expression of nine immunoblocking therapy associated genes did not have significant difference between Nrf2-inactivating MU and WT groups (Additional file 1: Fig. S6).

### Characteristics of TME in Nrf2-activating mutation group in the NSCLC cohort

**Fig. 8:**
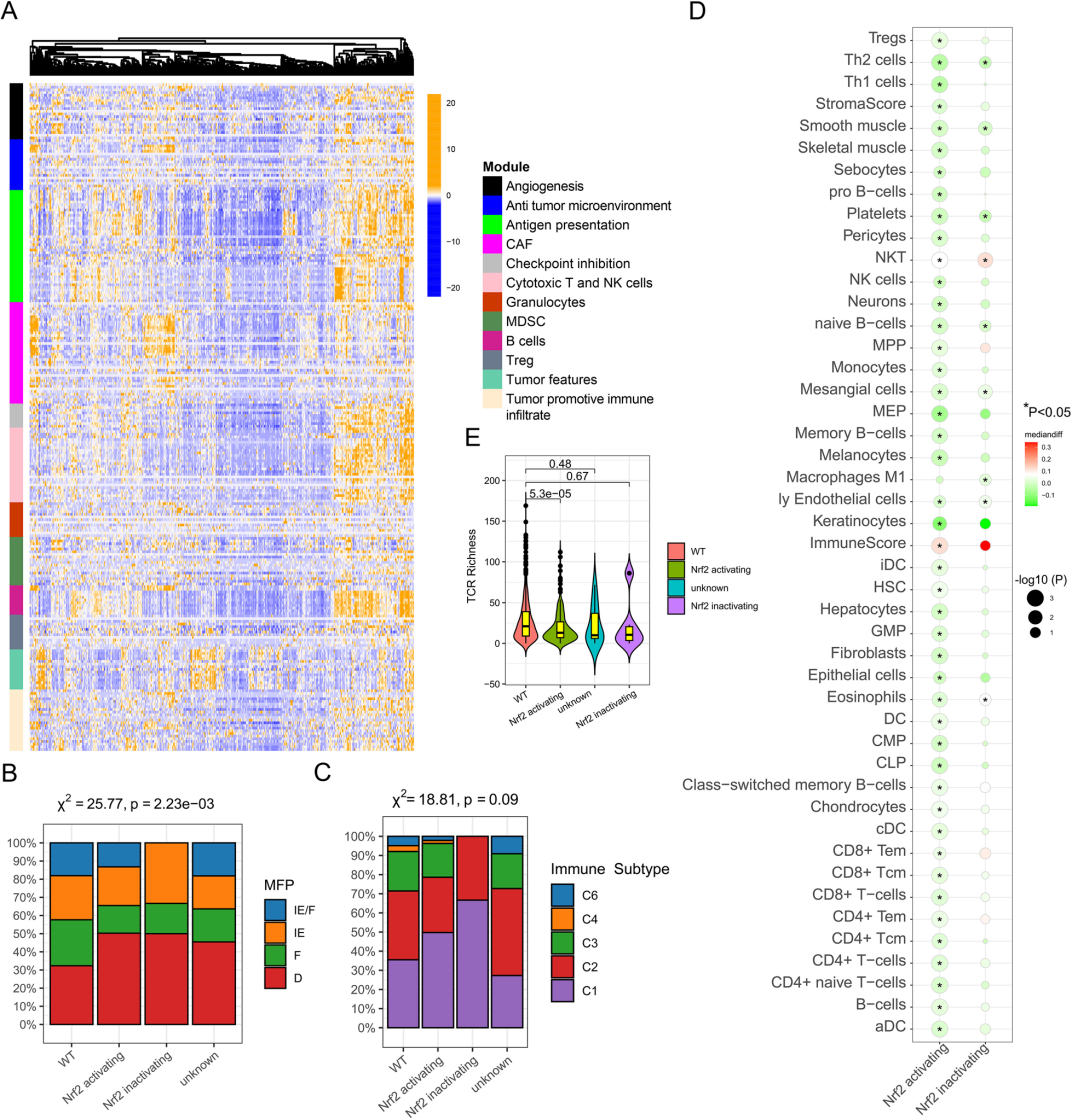
Landscape of the microenvironment phenotypes in NSCLC. (**A**) Heatmap representation of TME subtype Fges from TCGA NSCLC cohort using unsupervised hierarchical clustering. Distribution of Nrf2-activating MU and Nrf2-inactivating MU according to four TME subtypes (**B**) and five immune subtypes (**C**) in TCGA NSCLC cohort. (**D**) Immune deconvolution using xCell enriched in Nrf2-activating MU and Nrf2-inactivating MU patients with NSCLC. (**E**) Violin plots of TCR Richness among Nrf2-activating mutations, Nrf2-inactivating mutations, unknown NFE2L2 mutations and WT groups

Next, we analyzed the effect of Nrf2-activating MU on the TME using transcriptome data of 29 TME subtype Fges from TCGA NSCLC cohort. Unsupervised hierarchical clustering demonstrated that NFE2L2 mutated tumors have distinct immune-related transcriptome patterns (Fig. 8A) [53]. The distribution of four distinct TME subtypes (IE/F: immune-enriched, non-fibrotic; F: fibrotic; D: immune-depleted) were significantly different among four groups including Nrf2-activating MU, Nrf2-inactivating MU, unknown NFE2L2 MU and WT groups, with a more significant proportion of the D subtype observed in the Nrf2-activating MU and Nrf2-inactivating MU groups than those in the NFE2L2 WT group (Fig. 8B). But five immune subtypes (C1: wound healing; C2: IFN-g dominant; C3: inflammatory; C4: lymphocyte depleted; C5: immunologically quiet [missing]; C6: TGF-b dominant) did not have significant different among four groups (Fig. 8C) [54, 55]. This further indicated that immunosuppression occurred in Nrf2-activating MU NSCLC. Additionally, we used xCell to assess the TME cells and immunity function infiltrated using RNA-Sequencing data from Nrf2- activating MU and Nrf2-inactivating MU groups of tumor issue [56]. In Nrf2- activating MU group, there were fewer immune cells with dominant anti-tumor activity, such as Tregs, Th2 cells, Th1 cells, NK cells, Monocytes, T cells, CD8 + T cells, CD4 + T cells, and B cells (all p < 0.05, Fig. 8D). Notedly, compared to the NFE2L2 WT group TCR Richness and TCR Shannon were significantly decreased in Nrf2-activating MU group, not in Nrf2-inactivating MU group (Fig. 8E, Additional file 1: Fig. S7). The results suggested that Nrf2-activating MU might lead to immunosuppression in the tumor microenvironment of patients with NSCLC.

## Discussion

We addressed the landscape of NFE2L2 MUs and Nrf2-activating MUs and whether they impacted response/resistance to ICIs in NSCLC and other solid tumors. The result showed that: (i) the low frequency of NFE2L2 MUs (< 4%) was observed in the two pan-cancer cohorts with more common mutation cancers types including NSCLC, ESCA and head and neck cancer; (ii) The NFE2L2 MU and Nrf2-activating MU were associated with higher TMB value compared with wild-type in the two pan-cancer cohorts; (iii) OS were shorter in patients with NFE2L2 MU and Nrf2-activating MU compared with the WT group from NSCLC patients and pan-cancer patients in the MSK MetTropism cohort, LUAD patients in TCGA cohort; (iv) NFE2L2 MU and Nrf2-activating MU were not associated with OS in NSCLC patients and pan-cancer patients from several cohorts with ICIs treatment; (v) More objective responses to ICIs were observed in the patients with NFE2L2 MU than in those with NFE2L2 WT from several cohorts with ICIs treatment; and (vi) decreased inflammatory signaling pathways and immune-depleted immunological microenvironments were enriched in NSCLC patients with NFE2L2 MU and Nrf2-activating MU.

The NFE2L2 pathway plays a crucial role in cellular defense mechanisms against oxidative stress and the activation of antioxidant responses [26]. Typically, the protein Keap1 confines NFE2L2 within the cytoplasm [57]. Nevertheless, in the presence of oxidative stress or other external stimuli, the release of NFE2L2 occurs, leading to its translocation into the nucleus. Within the cellular nucleus, NFE2L2 engages in the formation of heterodimers alongside tiny Maf proteins, thereby establishing connections with antioxidant response elements (AREs) that are situated within the promoter regions of specific genes [58]. This interaction results in the initiation of transcriptional activity for a range of cytoprotective genes, encompassing those implicated in antioxidant defense, detoxification enzymes, and efflux transporters. The regulation of NFE2L2 activation is closely controlled through post-translational modifications and protein–protein interactions [59]. For example, the alteration of particular cysteine residues on Keap1 results in the separation of NFE2L2 and its subsequent movement into the cell nucleus. Moreover, it has been observed that NFE2L2 activity can be regulated by phosphorylation or other regulatory mechanisms through many signaling pathways, including the PI3K-AKT and MAPK pathways [60]. In addition, it should be noted that the NFE2L2 pathway exhibits interactions with many biological pathways and transcription factors, hence establishing intricate regulatory networks. As an illustration, the transcription factor NFE2L2 has the ability to engage in crosstalk with NF-κB, AP-1, and several other transcription factors in order to effectively regulate inflammatory responses and maintain cellular homeostasis [61]. The association between the interaction of KEAP1 and NFE2L2 has been implicated in the pathogenesis of various chronic conditions, such as diabetes, cancer, and neurodegenerative illnesses [11, 14, 19]. Furthermore, several clinical studies have demonstrated a notable correlation between NFE2L2 genetic mutations and worse prognosis in various types of malignancies, including LUSC, LUAD, liver hepatocellular carcinoma, and ESCA [23]. In accordance with prior studies, our findings also indicate a correlation between NFE2L2 MU and Nrf2-activating MU and unfavorable prognosis in our two comprehensive cancer datasets.

A previous study shows that high expression of nuclear NFE2L2 predicts poor prognosis in patients with esophageal squamous cell carcinoma [20]. Furthermore, high expression of NFE2L2 is positively correlated with the number of tumor-infiltrating lymphocytes, expression of immune checkpoint molecules and TME in the ER-Positive/HER2-Negative breast cancer [62]. However, this study noted decreased anti-tumor immunological microenvironments and decreased CTLA4 and PDCD1 expression in NSCLC patients with Nrf2-activating MU. Although an apparent immune-cold microenvironment in the NFE2L2 mutated NSCLC, durvalumab significantly improves local–regional control and reduces local–regional failure in NFE2L2 mutated patients with NSCLC after chemoradiation [63]. The situation in 133 pan-cancer patients is similar; ICI improves OS in patients with NFE2L2 MU compared with other treatments [23]. Another clinical study of 703 patients with advanced squamous NSCLC reveals that NFE2L2 MUs are associated with lower survival after platinum-based chemotherapy than the wild type, although this association is not evident in patients with NFE2L2 MUs who receive anti-PD-1/PD-L1 therapy [64]. As a result, despite NFE2L2 MUs may be associated with a cold immune environment, immunotherapy may still produce therapeutic benefits and prolong survival for tumor patients with NFE2L2 MUs.

In particular, when classifying Keap1/NFE2L2 mutations into different groups: Nrf2-activating MU, Nrf2-inactivating MU, unknown NFE2L2 MU and WT groups, it was observed that most immunotherapy-blocked genes were lower expressed, and there was a low enrichment of tumor-related immune cells, TCR richness, and Shannon diversity in Nrf2-activating MU group. However, such trends were not observed in the Nrf2-inactivating mutation group. These findings suggest that Nrf2-activating MU cancers, based on KEAP1/NFE2L2 mutations, may be more suitable for immunotherapy, might be more amenable to immunotherapy. While the presence of NFE2L2 mutations may result in a lower number of immune cells, there is potential for adoptive T cell therapy to enhance the efficacy of immunotherapy in NFE2L2 mutated cancers [65]. However, it is important to note that additional prospective clinical trials are necessary to validate these findings. Further research and investigation are required to determine the optimal therapeutic strategies and assess the impact of adoptive T cell therapy in patients with NFE2L2 mutations.

Inspired by several previous studies [31, 66–69], we explored the impact of NFE2L2 MUs on immunotherapy after adjusting the impacts of TMB and KEAP1 mutations. Although TMB was higher in NFE2L2 mutated patients than in wild-type patients in three pan-cancer cohorts, there was no statistically significant association between NFE2L2 MU and worse survival in the MSK1661 cohort. Likewise, a previous report revealed that increased TMB in LUSC, LUAD, COADREAD, GBAC and STAD, and NFE2L2 MU, is associated with a better prognosis [23]. Furthermore, interactions analysis revealed that TMB value, as well as other genes mutations, did not affect the association between NFE2L2 MU and OS, despite the fact that NFE2L2 MUs displaying the common co-occurrence with essential oncogenes such as TP53, TERT, CDKN2A, PBRM1, SETD2, EGFR, ATM, KEAP1 and STK11. This result was inconsistent with immunotherapy outcomes in LUAD and gastrointestinal cancer [9, 69–71]. This discrepancy can be attributable to the fact that earlier studies were limited to specific tumor types, whereas our analysis was based on multivariate COX regression of large pan-cancer samples to adjust for the co-mutation effect of other genes. Because NFE2L2 MUs are uncommon, a large enough sample size will be necessary in the future to determine whether they are independently associated with outcomes in other tumor types.

Activating mTORC1 signaling pathway accelerates the synthesis of proteins in cells to provide a material basis for tumor cell growth [72]. Thus, mTORC1 is an important target of cancer treatment. In this study, the mTORC1 signaling pathway was significantly activated in the NFE2L2 mutated patients with NSCLC, implying that TORC1 inhibitors were likely to exhibit considerable therapeutic efficacy in NFE2L2 mutated NSCLC patients. This finding appeared to be consistent with findings from a trial study in which TAK-228, a TORC1/2 inhibitor, demonstrated superior therapy efficacy in NFE2L2-mutated LUSC compared to KEAP1-mutated LUSC, KRAS/NFE2L2- or KEAP1-mutated NSCLC, despite the fact that the majority of patients received platinum-based chemotherapy and immunotherapy [73]. Thus, the NFE2L2 mutation can assist in classifying and identifying ICI patients, may have major implications for precision-targeted applications.

## Limitations

There are several limitations to our study. First, despite NFE2L2-mutated NSCLC being marked by changes in signaling pathways, such as in interferon response, inflammatory, JAK STAT3, p53, mTORC1, and ROS, the molecular basis of NFE2L2 MUs associated with ICI response remained unclear and required experimental research. Second, the low frequency of NFE2L2 MUs (1.9–3.6% in pan-cancers) might restrict the therapeutic use of detecting NFE2L2 MUs and explaining the mechanism of disease progression in NFE2L2 mutated tumors. Third, the large study included several cohorts with ICIs treatments, which might have sometimes caused in bias for data analysis. To a certain goal, we investigated the main clinical outcomes subtyped by study and tumor types, while a small sample size may lead to inadequate explanations for NEF2L2 MU effect.

## Conclusions

In summary, our study highlights the landscape of NFE2L2 MUs and Nrf2-activating MUs and their association with various aspects of cancer, including TMB, patient survival, response to ICIs, and the tumor microenvironment. These findings provide valuable insights into the complexity of Nrf2-activating MUs and their potential implications for cancer treatment, especially NSCLC. Further research and clinical trials are warranted to fully understand the underlying mechanisms and therapeutic strategies associated with NFE2L2 and Nrf2-activating mutations in NSCLC.

### Supplementary Information

**Additional file 1: Figure S1.** The flowchart of this study. The flowchart outlines the primary objective of the selected cohorts and the analytical procedure. **Figure S2.** Comparison of clinical characters among Nrf2-activating mutations, Nrf2-inactivating mutations, unknown NFE2L2 mutations and WT groups in the OrigiMed cohort. (**A**) The proportion of Nrf2-activating mutations, Nrf2-inactivating mutations and unknown NFE2L2 mutations. Distribution of tumor stage (**B**), metastasis status (**C**), sex (**D**) and treatment methods (**E**) among Nrf2-activating mutations, Nrf2-inactivating mutations, unknown NFE2L2 mutations and WT groups. (**F**) The correlation between the frequency of NFE2L2 mutation and objective response rates to ICIs stratified by cancer type. WT: wild type. **Figure S3.** Summary of NFE2L2 mutations, Nrf2-activating mutations and clinical characters in the MSK MetTropism cohort. (**A**) OncoPrint plot showing NFE2L2 MU across pan-cancers. (**B**) The proportion of Nrf2-activating mutations, Nrf2-inactivating mutations and unknown NFE2L2 mutations. (**C**) The correlation between the frequency of NFE2L2 mutation and median tumor mutation burden stratified by cancer types. Comparison of TMB (**D**), mutation count (**E**), MSI score (**F**), FGA (**G**) and metastasis status (**H**) among Nrf2-activating mutations, Nrf2-inactivating mutations, unknown NFE2L2 mutations and WT groups. (**I**) The correlation between the frequency of NFE2L2 mutation and objective response rates to ICIs stratified by cancer type. (**J**) Kaplan–Meier survival curves of OS between NFE2L2 MU and NFE2L2 WT groups. (**L**) Association of NFE2L2 mutation with OS stratified by cancer type. (**M**) Association of Nrf2-inactivating MU with OS stratified by cancer type. **Figure S4.** Summary of NFE2L2 mutations and survival outcomes in TCGA cohort and patients with NSCLC. (**A**) Association of NFE2L2 mutation with OS stratified by cancer type. (**B**) Kaplan–Meier survival curves of OS between NFE2L2 MU and NFE2L2 WT patients with NSCLC in TCGA cohort. (**C**) The multivariate Cox regression analysis for OS in patients with LUSC. **Figure S5. (A)** Meta-analysis for each cancer and melanoma to summarize association of NFE2L2 mutation with OS after ICIs treatment. Kaplan–Meier survival curves of OS between NFE2L2 MU and NFE2L2 WT NSCLC groups from the MSK1661 (**B**, n = 908), OAK (**C**, n = 324) and POPLAR (**D**, n = 105) cohorts. (**E**) Comparison of TMB between NFE2L2 MU and NFE2L2 WT NSCLC groups from the MSK1661, and POPLAR cohorts. (**F**) Kaplan–Meier survival curves of OS between HTMB and LTMB groups from the MSK1661, OAK and POPLAR cohorts. HTMB: high tumor mutation burden, L TMB: Low tumor mutation burden. (**G**) Kaplan–Meier survival curves of OS among HTMB NFE2L2 MU, HTMB NFE2L2 WT, LTMB NFE2L2 MU and LTMB NFE2L2 WT patients with NSCLC from the MSK1661, OAK and POPLAR cohorts. OS: overall survival; MU: mutation; WT: wild type. **Figure S6.** Several immune checkpoints block genes expression among Nrf2-activating mutations, Nrf2-inactivating mutations, unknown NFE2L2 mutations and WT groups with NSCLC in TCGA cohort. **Figure S7.** Violin plots of TCR Shannon among Nrf2-activating mutations, Nrf2-inactivating mutations, unknown NFE2L2 mutations and WT groups. **Table S1.** Summary of gene mutations concluded from NGS analysis.

## Data Availability

Genomic and transcriptomic sequence datasets in the OrigiMedm, MSK MetTropism and TCGA cohorts were downloaded from cBioPortal platform, seen in the Methods section. Other datasets including POPLAR and OAK cohorts, DFCI cohort, Pender cohort, and PUCH cohort are openly available and were downloaded from the following places: https://www.nature.com/articles/s41591-018-0134-3#MOESM3, https://www.nature.com/articles/s41588-018-0200-2#Sec24, https://www.cbioportal.org/study/summary?id=skcm_dfci_2015, https://www.bcgsc.ca/downloads/immunoPOG/ and https://doi.org/10.6084/m9.figshare.14168879. Data and materials can be provided upon reasonable request to the corresponding author.

## Abbreviations

ICB: Immune-checkpoint blocking
TMB: Tumor mutation burden
CNA: Copy number change
MSI: Microsatellite instability
TME: Immune microenvironment
NSCLC: Non-small cell lung cancer
LUSC: Lung squamous cell carcinoma
sqNSCLC: Squamous non-small cell lung cancer
OS: Overall survival
LUAD: Lung adenocarcinoma
NGS: Next-generation sequencing;
LIA: Clinical Laboratory Improvement Amendments
CAP: College of American Pathologists
DGE: Differential gene expression
GSEA: Gene set enrichment analysis
ESCA: Esophageal carcinoma
FGA: Fraction genome altered
