# Supplementary material for: Pan-cancer analysis of NFE2L2 mutations identifies a subset of lung cancers with distinct genomic and improved immunotherapy outcomes
Source: Cancer Cell Int. 2023 Oct 4;23:229. doi: 10.1186/s12935-023-03056-9 (PMC10552358; doi:10.1186/s12935-023-03056-9)
Supplement: Supplementary file 1 — Additional file 1: Figure S1. The flowchart of this study. The flowchart outlines the primary objective of the selected cohorts and the analytical procedure. Figure S2. Comparison of clinical characters among Nrf2-activating mutations, Nrf2-inactivating mutations, unknown NFE2L2 mutations and WT groups in the OrigiMed cohort. (A) The proportion of Nrf2-activating mutations, Nrf2-inactivating mutations and unknown NFE2L2 mutations. Distribution of tumor stage (B), metastasis status (C), sex (D) and treatment methods (E) among Nrf2-activating mutations, Nrf2-inactivating mutations, unknown NFE2L2 mutations and WT groups. (F) The correlation between the frequency of NFE2L2 mutation and objective response rates to ICIs stratified by cancer type. WT: wild type. Figure S3. Summary of NFE2L2 mutations, Nrf2-activating mutations and clinical characters in the MSK MetTropism cohort. (A) OncoPrint plot showing NFE2L2 MU across pan-cancers. (B) The proportion of Nrf2-activating mutations, Nrf2-inactivating mutations and unknown NFE2L2 mutations. (C) The correlation between the frequency of NFE2L2 mutation and median tumor mutation burden stratified by cancer types. Comparison of TMB (D), mutation count (E), MSI score (F), FGA (G) and metastasis status (H) among Nrf2-activating mutations, Nrf2-inactivating mutations, unknown NFE2L2 mutations and WT groups. (I) The correlation between the frequency of NFE2L2 mutation and objective response rates to ICIs stratified by cancer type. (J) Kaplan–Meier survival curves of OS between NFE2L2 MU and NFE2L2 WT groups. (L) Association of NFE2L2 mutation with OS stratified by cancer type. (M) Association of Nrf2-inactivating MU with OS stratified by cancer type. Figure S4. Summary of NFE2L2 mutations and survival outcomes in TCGA cohort and patients with NSCLC. (A) Association of NFE2L2 mutation with OS stratified by cancer type. (B) Kaplan–Meier survival curves of OS between NFE2L2 MU and NFE2L2 WT patients with NSCLC in TCGA cohort. (C) Th [file 12935_2023_3056_MOESM1_ESM.docx]

**Supplementary files**

**
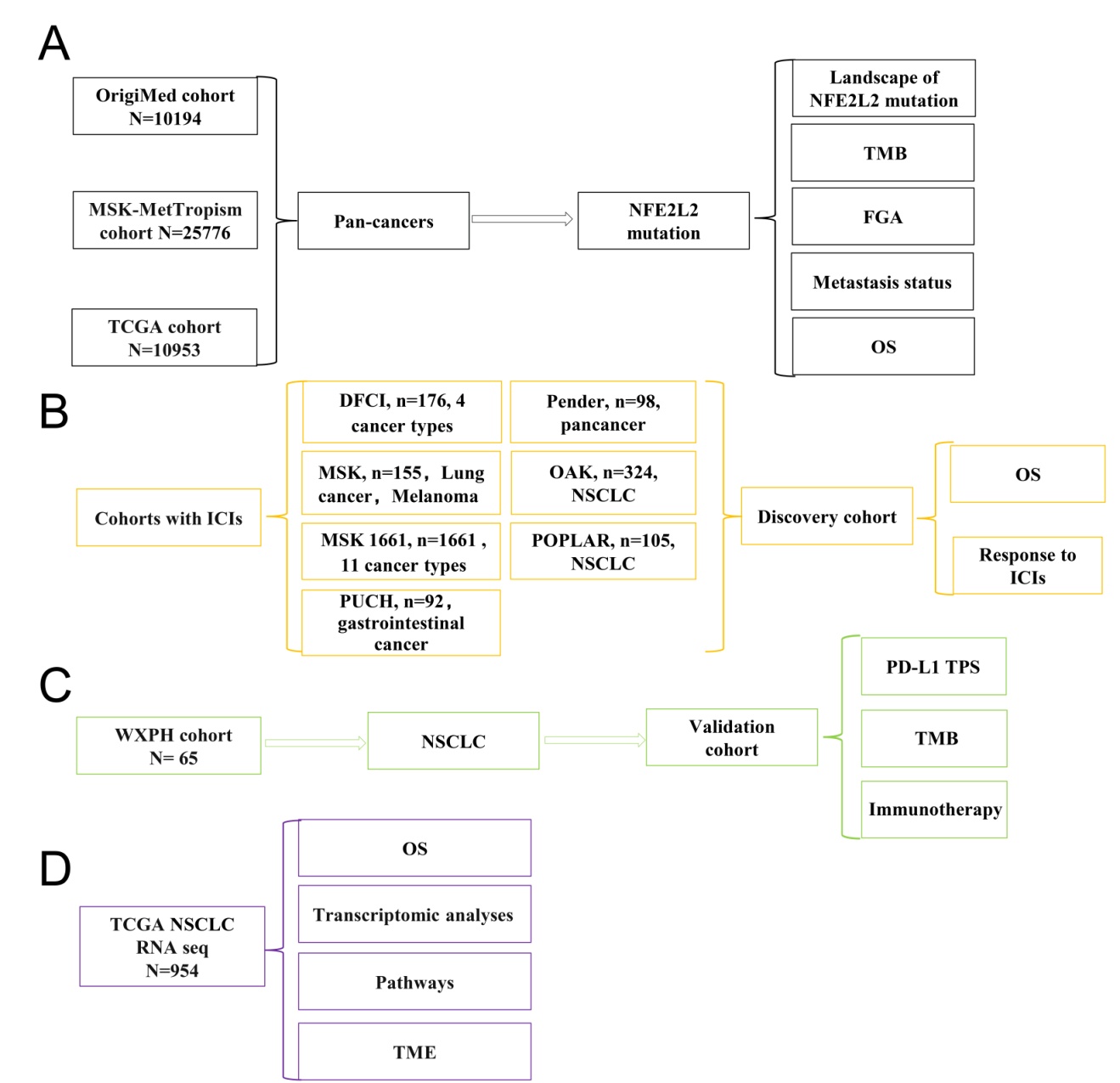
**

**Figure S1** The flowchart of this study. The flowchart outlines the primary objective of the selected cohorts and the analytical procedure.


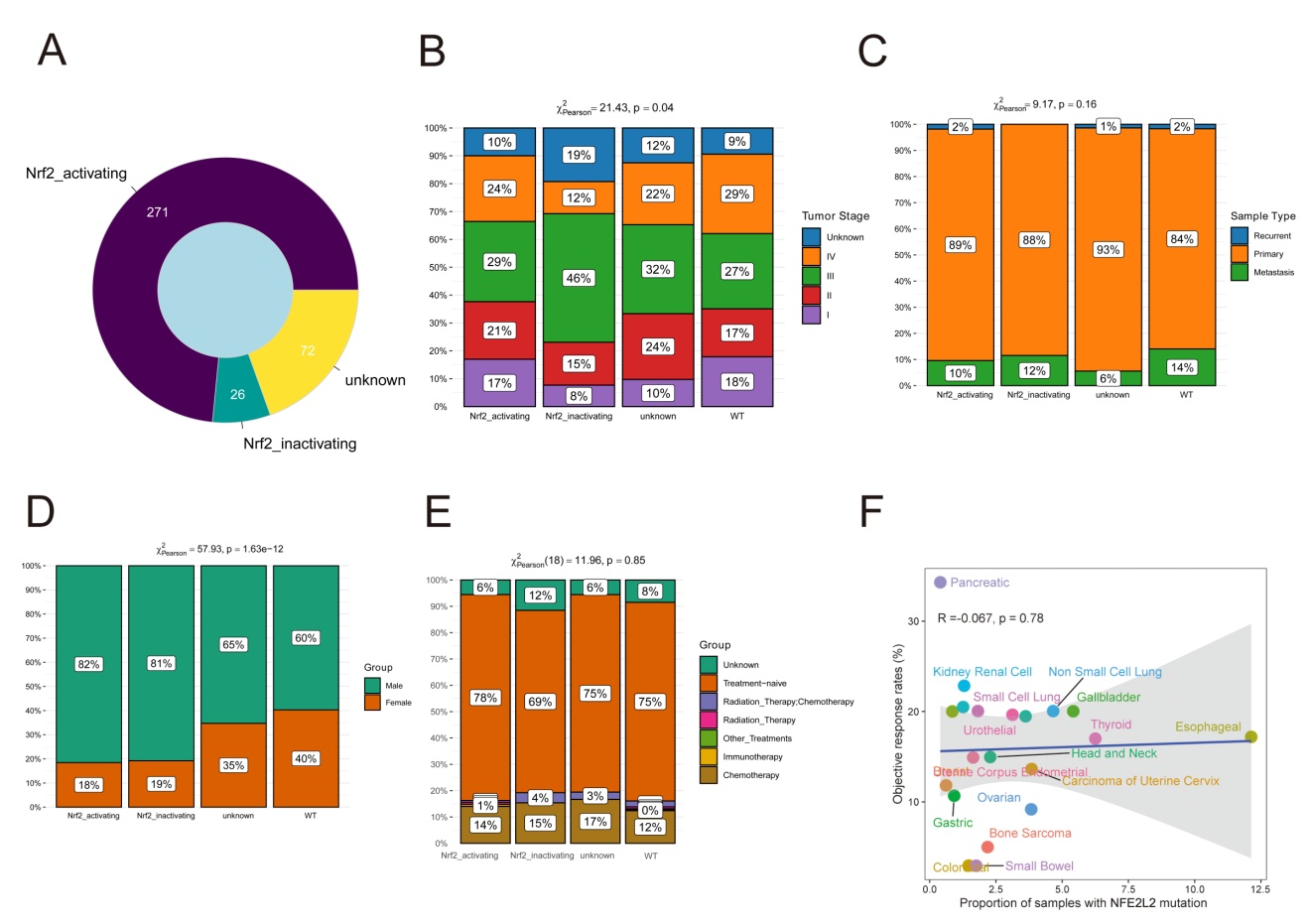


**Figure S2** Comparison of clinical characters among Nrf2-activating mutations, Nrf2-inactivating mutations, unknown NFE2L2 mutations and WT groups in the OrigiMed cohort. **A** The proportion of Nrf2-activating mutations, Nrf2-inactivating mutations and unknown NFE2L2 mutations. Distribution of tumor stage (**B**), metastasis status (**C**), sex (**D**) and treatment methods (**E**) among Nrf2-activating mutations, Nrf2-inactivating mutations, unknown NFE2L2 mutations and WT groups. **F** The correlation between the frequency of NFE2L2 mutation and objective response rates to ICIs stratified by cancer type. WT: wild type.


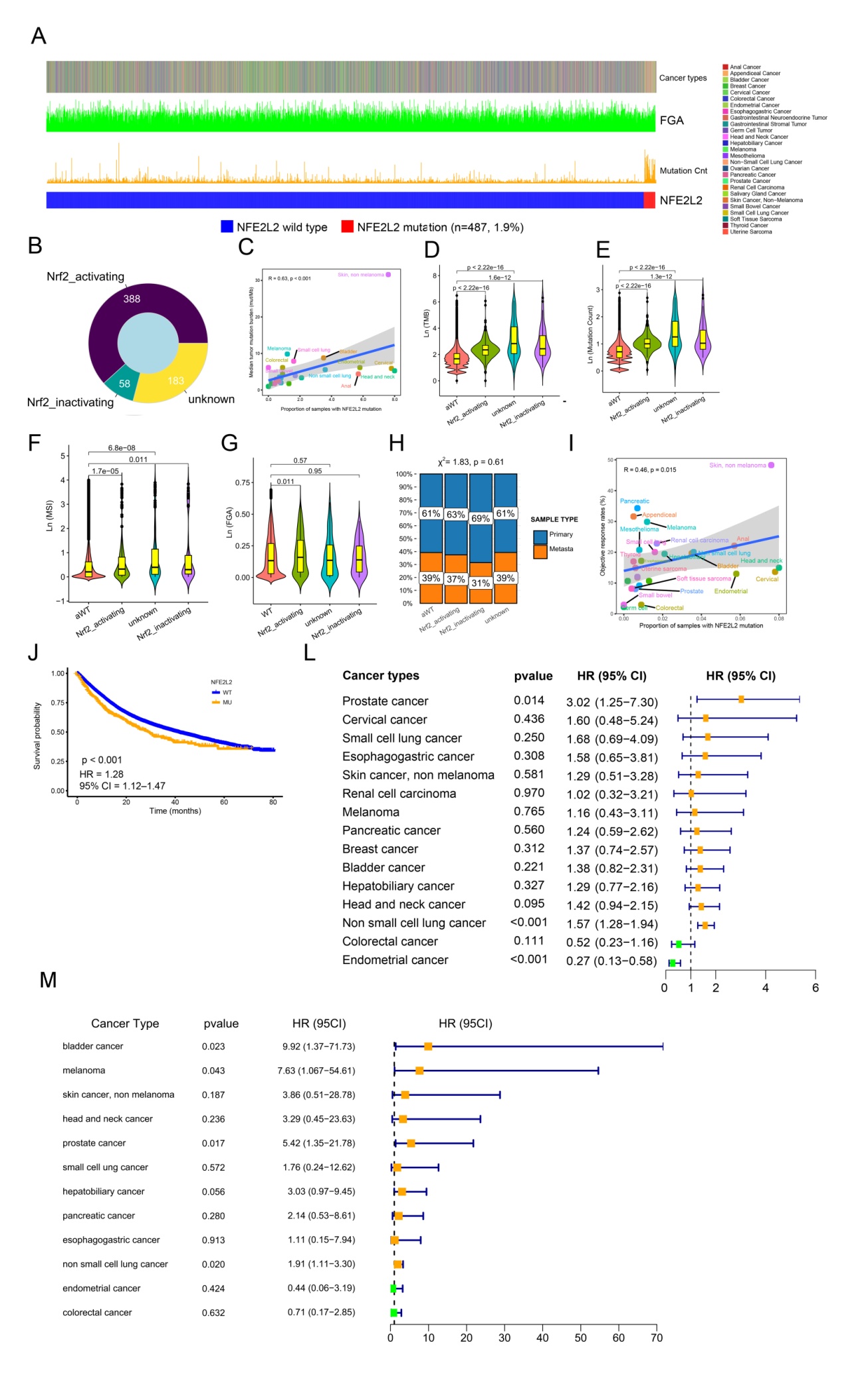


**Figure S3** Summary of NFE2L2 mutations, Nrf2-activating mutations and clinical characters in the MSK MetTropism cohort. **A** OncoPrint plot showing NFE2L2 MU across pan-cancers. **B** The proportion of Nrf2-activating mutations, Nrf2-inactivating mutations and unknown NFE2L2 mutations. **C** The correlation between the frequency of NFE2L2 mutation and median tumor mutation burden stratified by cancer types. Comparison of TMB (**D**), mutation count (**E**), MSI score (**F**), FGA (**G**) and metastasis status (**H**) among Nrf2-activating mutations, Nrf2-inactivating mutations, unknown NFE2L2 mutations and WT groups. **I** The correlation between the frequency of NFE2L2 mutation and objective response rates to ICIs stratified by cancer type. **J** Kaplan-Meier survival curves of OS between NFE2L2 MU and NFE2L2 WT groups. **L** Association of NFE2L2 mutation with OS stratified by cancer type. **M** Association of Nrf2-inactivating MU with OS stratified by cancer type.


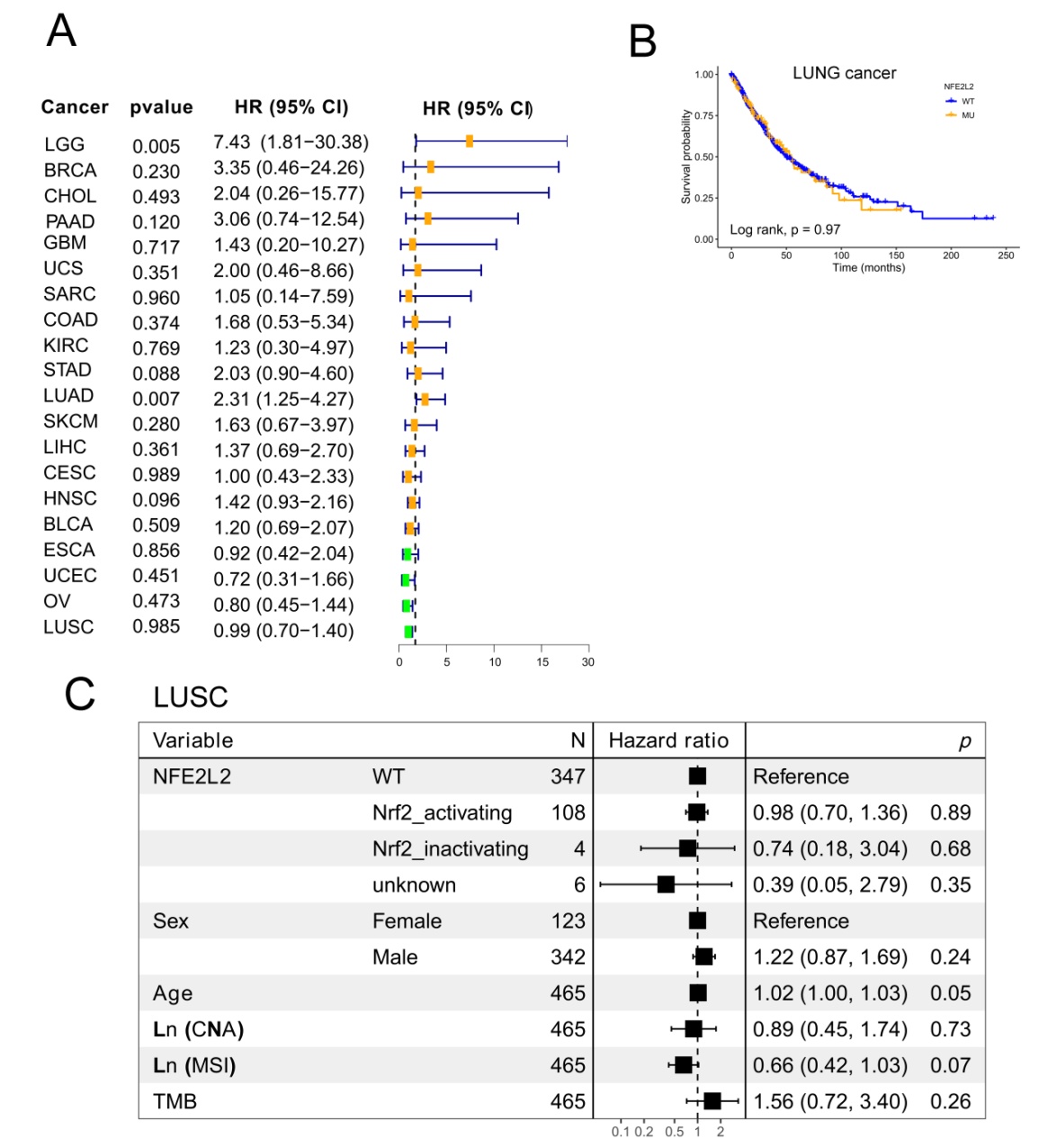


**Figure S4** Summary of NFE2L2 mutations and survival outcomes in TCGA cohort and patients with NSCLC. **A** Association of NFE2L2 mutation with OS stratified by cancer type. **B** Kaplan-Meier survival curves of OS between NFE2L2 MU and NFE2L2 WT patients with NSCLC in TCGA cohort. **C** The multivariate Cox regression analysis for OS in patients with LUSC.


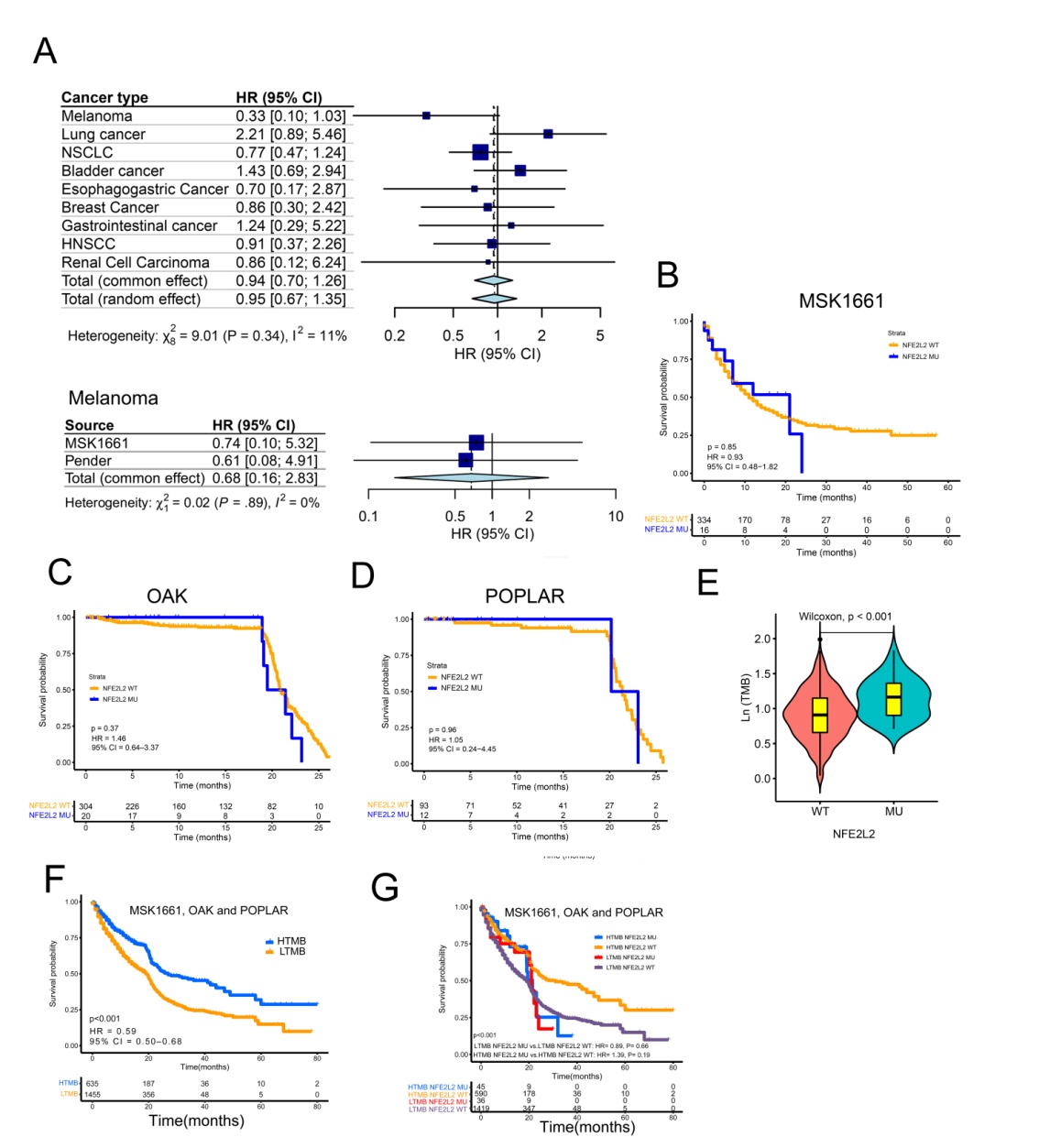


**Figure S5 A** Meta-analysis for each cancer and melanoma to summarize association of NFE2L2 mutation with OS after ICIs treatment. Kaplan-Meier survival curves of OS between NFE2L2 MU and NFE2L2 WT NSCLC groups from the MSK1661 (**B**, n=908), OAK (**C**, n=324) and POPLAR (**D**, n=105) cohorts. **E** Comparison of TMB between NFE2L2 MU and NFE2L2 WT NSCLC groups from the MSK1661, and POPLAR cohorts. **F** Kaplan-Meier survival curves of OS between HTMB and LTMB groups from the MSK1661, OAK and POPLAR cohorts. HTMB: high tumor mutation burden, L TMB: Low tumor mutation burden. **G** Kaplan-Meier survival curves of OS among HTMB NFE2L2 MU, HTMB NFE2L2 WT, LTMB NFE2L2 MU and LTMB NFE2L2 WT patients with NSCLC from the MSK1661, OAK and POPLAR cohorts. OS: overall survival; MU: mutation; WT: wild type.


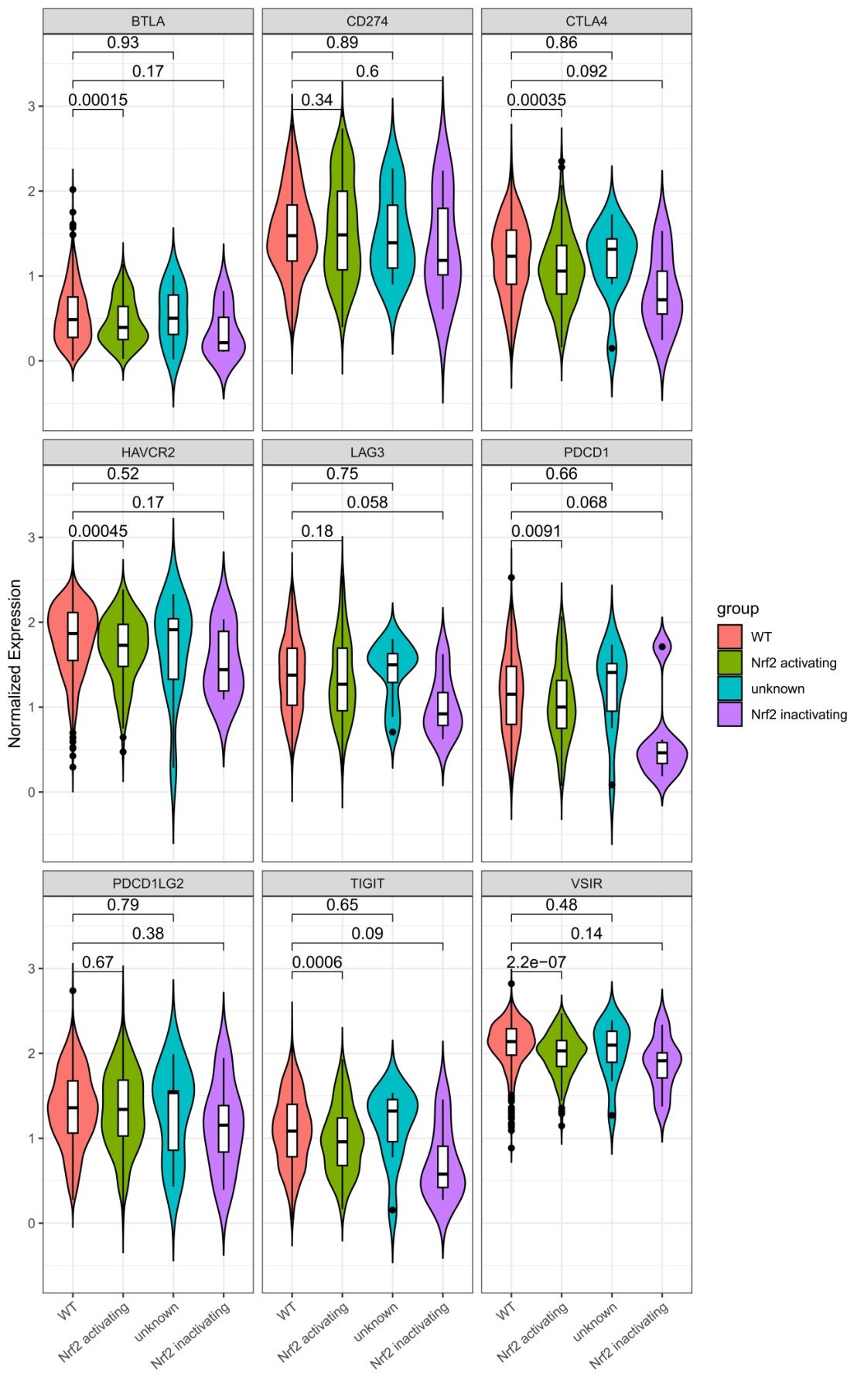


**Figure S6** Several immune checkpoints block genes expression among Nrf2-activating mutations, Nrf2-inactivating mutations, unknown NFE2L2 mutations and WT groups with NSCLC in TCGA cohort.


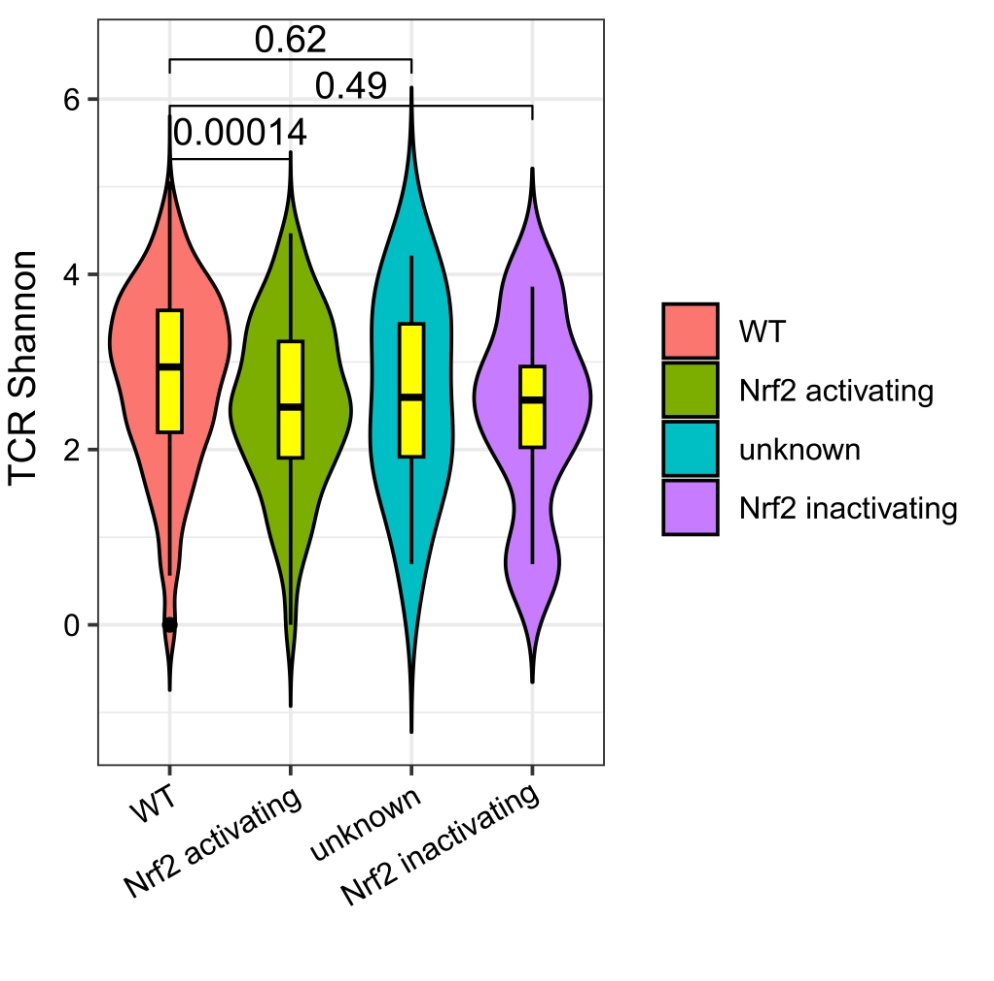


**Figure S7** Violin plots of TCR Shannon among Nrf2-activating mutations, Nrf2-inactivating mutations, unknown NFE2L2 mutations and WT groups.

**Table S1 Summary of gene mutations concluded from NGS analysis.**

| **gene** | **variation type** | **cDNA variation** | **abundance** |
| --- | --- | --- | --- |
| STK11 | alternative splicing | c.598-1G>A | 42.61% |
| KEAP1 | missense | c.1714T>G | 39.45% |
| TP53 | nonsense | c.298C>T | 34.21% |
| IFNGR1 | missense | c.1445C>T | 16.85% |
| EPHA3 | missense | c.2525G>T | 16.39% |
| LRP1B | missense | c.4129A>G | 14.88% |
| SPTA1 | missense | c.5821C>G | 11.02% |
| CDK6 | amplification | - | CN:3.7 |
| NFE2L2 | amplification | - | CN:4.1 |
| CCND3 | amplification | - | CN:4.3 |
| PTPRD | amplification | - | CN:4.3 |
| TRRAP | amplification | - | CN:4.7 |
| JAK2 | amplification | - | CN:5.4 |
| TERT | amplification | - | CN:5.8 |
| CD274 | amplification | - | CN:6.1 |
| SDHA | amplification | - | CN:8.4 |
